# Phytochemistry, Chemotaxonomy, and Biological Activities of the Araucariaceae Family—A Review

**DOI:** 10.3390/plants9070888

**Published:** 2020-07-14

**Authors:** Claudio Frezza, Alessandro Venditti, Daniela De Vita, Chiara Toniolo, Marco Franceschin, Antonio Ventrone, Lamberto Tomassini, Sebastiano Foddai, Marcella Guiso, Marcello Nicoletti, Armandodoriano Bianco, Mauro Serafini

**Affiliations:** 1 Dipartimento di Biologia Ambientale, Università di Roma “La Sapienza”, Piazzale Aldo Moro 5, 00185 Rome, Italy; daniela.devita@uniroma1.it (D.D.V.); chiara.toniolo@uniroma1.it (C.T.); antonio.ventrone@uniroma1.it (A.V.); lamberto.tomassini@uniroma1.it (L.T.); sebastiano.foddai@uniroma1.it (S.F.); marcello.nicoletti@uniroma1.it (M.N.); mauro.serafini@uniroma1.it (M.S.); 2 Dipartimento di Chimica, Università di Roma “La Sapienza”, Piazzale Aldo Moro 5, 00185 Rome, Italy; alessandro.venditti@gmail.com (A.V.); marco.franceschin@uniroma1.it (M.F.); marcella.guiso@uniroma1.it (M.G.); armandodoriano.bianco@uniroma1.it (A.B.)

**Keywords:** Araucariaceae, phytochemistry, ethnopharmacology, chemotaxonomy, biological activities

## Abstract

In this review article, the phytochemistry of the species belonging to the Araucariaceae family is explored. Among these, in particular, it is given a wide overview on the phytochemical profile of *Wollemia* genus, for the first time. In addition to this, the ethnopharmacology and the general biological activities associated to the Araucariaceae species are singularly described. Lastly, the chemotaxonomy at the genus and family levels is described and detailed.

## 1. Introduction

Araucariaceae Henkel and W. Hochstetter is a family of coniferous trees, classified under the order Pinales, the class Pinopsoda, the division Pinophyta, and the Clade Tracheophytes [1]. 

It is a very ancient family since its maximum diversity was achieved during the Jurassic and Cretaceous periods with a worldwide distribution. Yet, during the extinction events occurred in the transition from Cretaceous to Paleogene, these species totally vanished from the Northern Hemisphere whereas they remained in the Southern Hemisphere apart for a very few exceptions. In particular, Araucariaceae species are well present in South America, Australia, New Zealand, New Guinea, New Caledonia, and other South Pacific islands while Malaysia represents the exception [2]. 

From the taxonomic point of view, the family comprises four genera: *Agathis* Salisb., *Araucaria* Juss., *Columbea* Salisb., and *Wollemia* W.G.Jones, K.D.Hill and J.M.Allen. Yet, *Columbea* and *Wollemia* genera are formed by one only species each i.e., *Columbea brasiliensis* (A. Rich.) Carrière and *Wollemia nobilis* W.G.Jones, K.D.Hill and J.M.Allen. On the other hand, *Agathis* genus is formed by 18 accepted species and 4 unresolved species whilst *Araucaria* genus is formed by 19 accepted species [3]. 

From the phylogenetic standpoint derived from molecular data, Araucariaceae family belongs to a major subdivision that includes the Podocarpaceae, Sciadopityaceae, Cupressaceae, Cephalotaxaceae, and Taxaceae families. In particular, Araucariaceae family belongs to the same clade as Podocarpaceae and represents the least evolved family of the subdivision. Within the family itself, the phylogeny tree forecasts *Wollemia* genus as the least evolved one followed by *Agathis* and *Araucaria* genera. Within the *Araucaria* genus, the situation is more complex, with several existing sub-clades [1]. 

In the following pages, all the general botanical features, the phytochemistry, the ethnopharmacology, and the biological activities associated to each genus are described separately. The following databases were used for this study: Scopus, Google Scholar, PubMed, Reaxys, SciFinder, PubChem. The literature research was conducted by digiting every single species name as reported in the site ww.theplantlist.org [3] in all the databases and collecting the relative outcome data.

## 2. Genus *Agathis*

### 2.1. Botany

**Figure 1:**
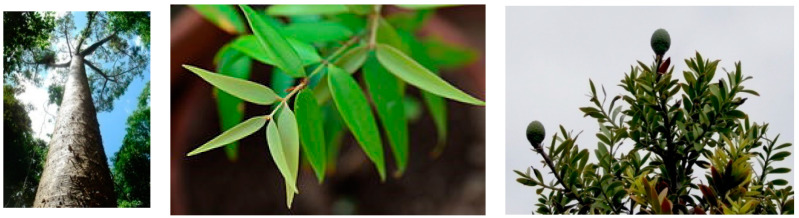
Images of the organs of *Agathis* species: *A. microstachya* trunk (**left**); *A. philippinensis* leaves (**middle**); *A. australis* leaves and cones (**right**).

The species belonging to this genus are usually monoecious. They are characterized by a large and very robust trunk with no branching in the inferior part when they are mature trees. Indeed, when they are young, they are generally conical and have more irregular crowns. The bark is smooth and grey-brownish colored, usually with a peeling that form irregular flakes that become thicker and thicker as the age of the tree proceeds. The branches are often horizontal, or ascending when they are too large. The lower ones often leave circular branch scars when they detach from the lower trunk. The juvenile leaves are larger than the adult ones and are more or less acute, with an ovate to lanceolate shape. They are often coppery-red colored also. Indeed, the adult leaves are opposite, from linear to elliptical, quite thick and very leathery. They produce two cones: male (pollen) and female (seed). The male ones appear only on the largest trees. The female ones usually develop on short lateral branchlets and get mature after two years. They are generally oval or globe shaped (Figure 1) [4].

### 2.2. Distribution

*Agathis* is a quite widespread genus of the family. In fact, their species can be found in New Zealand, the Philippines, New Guinea, Melanesia, and Australia, but also in Malaysia, beyond the Equator line. They grow on diverse substrates including podzolized sands, ultramafics, carbonates, and silicates. They occur from near sea level to the altitude of about 2500 m. They mainly prefer sites that never see frost, and that receive between five and ten meters of rain per year [5].

### 2.3. Phytochemistry

Table 1 shows data of all the compounds identified in the genus divided according to the species.

As Table 1 clearly shows, not all the existing species of the genus have been studied. In addition, most of the phytochemical works reported in the literature about this genus regards species collected in Oceania or in South-Eastern Asia [8,10,11,12,21,24] except two, whose studied exemplars were collected in Italy and United Kingdom and these are both associated with *A. robusta* [22,23]. This fact is not so unusual since, as already mentioned, these species are mainly known to grow in those areas. Nevertheless, only about the exemplar collected in Italy, the phytochemical characterization has been fully described and the reported compounds are quite similar to those reported for the other samples collected in other growth areas. Yet, in order to verify if this is a general tendency, more phytochemical studies must be carried out both on the same exemplar and on other samples coming from different areas of Italy and of the world. In addition, in all the cases, more exemplars coming from different areas were studied [8,9,10,12,13,16,17,18,19,22,23,24,25] except for *A. borneensis* samples coming only from Malaysia [11] and *A. microstachya* samples coming only from Australia [8,20]. For what concerns the studied organs, leaves represent the most studied ones. Nevertheless, resin, stem barks, branches, and the generic aerial parts have also been considered. In some cases, one only type of organs were analyzed i.e., *A. alba*, *A. australis*, *A. dammara*, and *A. ovata* of which only the leaves were analyzed [6,7,8,9,12,13], *A. lanceolata* of which only the phytochemical analysis of the resin is reported in literature [14,15] and *A. moorei* of which only the phytochemical analysis of the leaves is reported in literature [8]. The exudate of only *A. philippinensis* [21] as well as the seeds of only *A. robusta* purchased in the United Kingdom [25] have been analyzed for their phytochemical composition reporting the presence of essential oil metabolites for the former and fatty acids for the latter. Right about this point, essential oil components and polar fraction components have been evidenced in the genus. Yet, only for six species i.e., *A. atropurpurea* [8,9], *A. australis* [8,9], *A. macrophylla* [8,16,17,18,19], *A. microstachya* [8,20], *A. ovata* [8,9] and *A. robusta* [8,9,22,23,24,25], the phytochemical studies regarded both kinds of natural compounds. Indeed, for four species i.e., *A. borneensis* [11], *A. dammara* [12], *A. moorei* [8], and *A. philippinensis* [21], only the essential oil composition was studied whereas for two species i.e., *A. alba* [6,7] and *A. lanceolata* [14,15], only the polar fraction composition was analyzed. Among the essential oil metabolites, none has been reported in all the compositions present in literature. Yet, 16-kaurene, α-copaene, α-cubebene, α-pinene, β-caryophyllene, β-pinene, δ-cadinene, *allo*-aromadendrene, aromadendrene, camphene, germacrene D, limonene, myrcene, sabinene, and spathulenol represent the most common compounds in this context. Among them, none can actually be used as chemotaxonomic marker at the genus level since they are quite widespread compounds as constituents of the plant essential oils [26,27]. Additionally, among the polar fraction metabolites, none have been reported in all the compositions present in literature. Yet, biflavonoids and, in particular, agathisflavone and its derivatives represent the most common compounds in this context. By the way, these compounds have actually been used as chemotaxonomic markers at the genus level [22,28,29,30,31,32,33], even if their occurrence seems, now, to be extended at the whole family level. Some diterpenes have also been reported for this genus from *A. lanceolata* [14,15], *A. macrophylla* from China [16] and from Fiji [18,19] and the resin of *A. microstachya* [20]. Only in one case i.e., *A. macrophylla*, some triterpenes have also been noticed [17]. In two cases i.e., *A. dammara* from Philippines [13] and *A. robusta* from United Kingdom [23], the exact polar fraction composition was not reported since only a phytochemical screening was performed. In both cases, the presence of flavonoids, tannins and phenolics has been reported. For what concerns the methodology, in some cases, the essential oil was obtained through hydrodistillation [10,12,21,24] whereas for three cases, solvent extraction [11,25] and solvent distillation [8] were used. In all the cases, multiple and different GC analyses were used for the separation and identification of the essential oil metabolites [8,10,11,12,21,24,25]. In this regard it should be underlined the presence of improbable natural products, such as iodo-derivatives, and a possible artifact, methyl-β-D-mannofuranoside, due to the extraction solvent [34], among the constituents identified by Adam and colleagues in *A. borneensis* [11]. In some cases i.e., *A. atropurpurea* leaves from Australia [8], *A. australis* leaves [8], *A. macrophylla* from Australia [8], *A. microstachya* leaves [8], *A. moorei* [8], *A. ovata* from Australia [8], and *A. robusta* from Australia [8], [α]_D_ and NMR analyses accompanied the GC ones. Indeed, for the analysis of the polar fraction metabolites, SE was the only method for the extraction of these compounds, CC, TLC, LC, and HPLC, together or separated, were the methods for the separation procedure of the compounds and [α]_D_, IR, NMR, and MS, together or separated, were the methods for the identification procedure of the compounds [6,7,9,14,15,16,17,18,19,20,22].

### 2.4. Ethnopharmacology

The ethnopharmacological uses of species belonging to the *Agathis* genus are quite limited. In fact, only a couple of works have dealt with this argument. In particular, it was reported that, in Borneo, *A. borneensis* is used to treat fever by boiling the bark in water and drinking it as an herbal tea [35]. Moreover, still in Borneo, the powdered woods of *A. borneensis*, *A. philippinensis* and *A. dammara* are employed to treat headache and myalgia [36].

### 2.5. Biological Activities

#### 2.5.1. Extracts

The biological activities associated with the essential oil or the extracts of *Agathis* species are quite numerous.

*A. atropurpurea* resin extract has shown to possess medium antifungal properties against *Aspergillus niger* and *Rhizopus stolonifer* with MIC values equal to 625 and 1250 μg/mL, respectively [37]. It also showed strong antileishmanial activities against *L. amazonensis* promastigotes and amastigotes with IC_50_ values equal to < 12.5 and 19.3 μg/mL, respectively, as well as weak cytotoxic effects against BALB/c mouse macrophage cells with a CC_50_ value equal to 118.4 μg/mL [38].

*A. borneensis* leaf methanol extract is able to exert strong antiplasmodial properties against *Plasmodium falciparum* D10 strain (sensitive strain) with an IC_50_ value equal to 11.00 μg/mL [36].

The essential oil of *A. dammara* exerts good antibacterial effects against several bacterial strains (*Staphylococcus aureus*, *Bacillus subtilis*, *Pneumonia aeruginosa*, and *Escherichia coli*) with inhibition zones in the range of 14.5–23.7 mm and with MIC values ranging from 1.25 to 2.5 mg/mL [12]. In addition to this, the methanolic extract obtained from its leaves was found to be active also against *Proteus vulgaris* with an inhibition zone in the range 19–21 mm [13]. Moreover, the *n*-hexane, methanol, and ethyl acetate extracts of its resin display very low antioxidant effects (IC_50_ values equal to 438.55, 313.51, 245.99 mg/mL, respectively) [39].

The hydroalcoholic extract of *A. robusta* from England at the concentration of 400 μg/mL has shown good anti-inflammatory activities according to the HRBC (HumanRedBloodCell) membrane stabilization and heat-induced hemolytic methods with percentages of denaturation inhibition equal to 76.84% and 77.12%, which are comparable to those observed for diclofenac (79.25%) and aspirin (83.78%) for the respective models [23]. Moreover, the resin essential oil has shown interesting antibacterial effects against several bacterial strains (*Staphylococcus* spp., *Klebsiella pneumoniae*, *Escherichia coli*, *Salmonella typhimurium* and *Pseudomonas aeruginosa*) with MIC and MBC values ranging from 250 to 500 and from 500 to more than 1000 μg/mL, respectively [24].

#### 2.5.2. Phytochemicals

In literature there are also some works about the biological activities associated with specific compounds isolated from *Agathis* species.

3-oxo-podocarp-8(14)-en-19-oic acid, 16-hydroxy-8(17),13-labdadien-15,16-olid-19-oic acid, 15ξ-hydroxypinusolidic acid and lambertianic acid isolated from *A. macrophylla*, are time-dependent moderate inhibitors of tyrosine phosphatase 1B (PTP1B) with k_i_ values of 0.11, 0.07 and 0.058 M^−1^s^−1^, respectively [16]. Moreover, (4*S*,5*R*,9*S*,10*R*)-methyl-19-hydroxy-15,16-dinorlabda-8(17),11-*E*- dien-13-oxo-18-oate, (4*R*,5*R*,9*R*,10*R*,13*S*)-13-hydroxypodocarp-8(14)-en-19-oic acid, (4*R*,5*R*,9*R*,10*R*,13*R*)-13-hydroxypodocarp-8(14)-en-19-oic acid, 15-*nor*-14-oxolabda-8(17),12*E*-dien-19-oic acid, 13-oxo-podocarp-8(14)-en-19-oic acid, 13-oxo-podocarp-8(14)-en-19-oate, 16-hydroxy-8(17),13-labdadien-15,16-olid-19-oic acid, 15ξ-hydroxypinusolidic acid, lambertianic acid, methyl lambertianate, pinusolidic acid, pinusolide, angustanoic acid F and 8,11,13-abietatrien-15-ol, again isolated from *A. macrophylla*, showed quite weak anticancer properties against HL-60 (human promyelocytic leukemia) and SMMC-7721 (human hepatocarcinoma) cancer cell lines [16].

7α,15α-dihydroxystigmast-4-en-3-one and 3β,22,23-trihydroxystigmast-5-en-7-one, isolated from *A. macrophylla*, have shown medium cytotoxic effects against A549 (adenocarcinomic human alveolar basal epithelial) tumor cell lines with IC_50_ values equal to 36.5 and 16.0 μmol/L [17].

### 2.6. Other Facts

In literature some interesting curiosities about *Agathis* species are also reported. In particular, these curiosities regard other uses of these species in the past.

*Agathis* spp. have been widely used for their timber in order to make panels, cabinets, joinery, turnery, moldings, patterns making, battery separators, piano parts, and artificial limbs. This is because the timber is straight-grained, strong, knot-free, with a silky and lustrous surface and it can be easily worked. In addition, the resin has been used to make the so-called Manila copal to be a component of varnishes, mainly. This resin derives from their living inner barks, is translucent or clear white, and slowly hardens on exposure to air with age, eventually becoming brittle. It is soluble in alcohol to a varying degree and has a melting point between 115–135 °C. Actually, this copal is a complex mixture of monoterpenes, sesquiterpenes, and diterpenes [40].

The young gum of *A. australis* has been used for many centuries as chewing gum by Maori people [41].

The Borneo aborigens consider *A. boorneensis* as a magical plant, capable to exert special powers including protection against bad spirits.

## 3. Genus *Araucaria*

### 3.1. Botany

**Figure 2:**
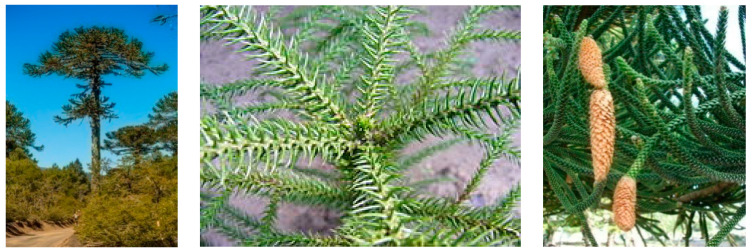
Images of the organs of *Araucaria* species: *A. araucana* tree (**left**), *A. heterophylla* leaves (**middle**), *A. columnaris* cones (**right**).

The species belonging to this genus are mostly dioecious trees even if monoecious trees can also be found. Moreover, some trees are even able to change sex with time. These species are characterized by a massive and erect stem. They can reach up to 80 m high. The branches are gathered in whorls and grow horizontally. They are covered with leathery or needled leaves, with no branching in the inferior part when they are mature trees. In some species, the leaves are narrow and lanceolate, barely overlapping each other, whilst in others they are very broad, flat, and widely overlapped with each other. They produce two cones: male (pollen) and female (seed). The female cones are globose and can be very variable size according to the species. They are found only on the top of the tree and they contain many large edible seeds. Indeed, the male cones are smaller in size and present a broad cylindrical shape (Figure 2) [42].

### 3.2. Distribution

*Araucaria* is widespread only in the Southern Hemisphere even if at different meridians. In fact, their species can be found in New Guinea, Australia, but also in Chile, Argentina, Brazil, New Caledonia, and Norfolk Island. In addition to this, there is a naturalized population of *A. columnaris*(G.Forst.) Hook. on the island of Lanai in the Hawaii. The greatest biodiversity of the genus is in New Caledonia. They prefer ultrabasic schistose and calcareous soils [43].

### 3.3. Phytochemistry

Table 2 shows data of all the compounds identified in the genus divided according to the species.

As Table 2 clearly shows, not all the existing species of this genus have been studied, too. For what concerns the collection sites of the studied species, most of the phytochemical works reported regard species collected in Oceania, South-Eastern Asia, or Southern America [6,8,44,45,46,47,48,49,50,51,52,53,54,55,56,57,58,59,60,61,62,64,67,70,71,74,75,76,77,78,79,80,81,82,87,88]. Only in a few cases, the phytochemical works regarded species collected in other areas of the world such as Europe and Africa [63,68,69,72,73,83,84,85,86]. This fact is, again, not so unusual since, as already mentioned, these species are mainly known to grow in those areas even if *Araucaria* species are, anyway, more widespread than all the other genera of this family. In one further case, the collection site of the studied species could not be obtained [66] whereas in the case of *A. columnaris*, it was purchased and not collected in the wild [78]. For what concerns the studied organs, leaves remain the most studied ones [6,8,63,64,68,69,70,76,82,83,85,87,88]. Nevertheless, many other organs have been considered for their phytochemical constituents for this genus. In particular, these organs are: the generic aerial parts [78,79], the foliage [81], the bark [44,74], the bracts [45], the branches [65,75], the cells [57], the dead bark [46], the female strobili [48], the knots [50,51], the needles [52,53,73], the oleoresin [71,72], the resin [54,58,59,60,61,67], the resin oil [81], the resin from the stems [86], the seeds [47,55], the stem bark resin [84], the whole plant [56,77], and the wood [62]. In one case i.e., *A.cunninghamii* from India, the study on the foliage was further divided into fresh and senescent foliage [81], and in one further case i.e., *A. columnaris* from China, twigs and leaves were studied together [80]. Lastly, in one only case, none could be obtained about the studied organs i.e., *A. araucana* [66]. For what concerns the reported phytochemical metabolite composition, both essential oil and polar fractions metabolites were observed. Both compositions were analyzed in most cases. Indeed, in a few cases, only the essential oil composition was analyzed such as for *A. hunsteinii*, *A. luxurians*, *A. montana*, *A. muelleri* and *A. scopulorum* [8] whereas in one only case i.e., *A. rulei*, only the polar fraction composition was studied [89]. Anyway, in no case, the same plant exemplar was used to study both the essential oil and polar fraction compositions. In other cases, the phytochemical studies were only phytochemical screenings reporting the classes of the natural compounds present like for the cooked seeds of *A. angustifolia* from Brazil [47], the oleoresin of *A.bidwilli* from India [71], the whole plant of *A. columnaris* from India [74], the aerial parts of *A. columnaris* from Pakistan [78], the stem bark resin of A. *cunninghamii* from South Africa [84], the leaves of *A. heterophylla* from Egypt [85], the whole plant of *A. heterophylla* from India [77] and the leaves of *A. heterophylla* from Indonesia [88]. In a few cases, the phytochemical composition was given only partially such as for the needles and the seeds of *A. angustifolia* from Brazil [52,53,55], the leaves of *A. bidwilli* from India [6,70], the fresh and senescent foliage, the resin oil and the leaves of *A. cunninghamii* from India [70,81], the foliage and the resin oil *A. heterophylla* from India [70,81]. Among the essential oil metabolites, none has been reported in all the compositions present in literature. Yet, 16-kaurene, α-copaene, α-cubebene, α-pinene, β-caryophyllene, β-pinene, δ-cadinene, *allo*-aromadendrene, aromadendrene, camphene, caryophyllene oxide, germacrene D, globulol, hibaene, humulene, limonene, luxuriadiene, myrcene, *p*-cymene, phyllocladene, spathulenol, viridiflorene, and viridiflorol represent the most common compounds in this context. Among them, none can again be used as chemotaxonomic marker at the genus level since they are quite widespread compounds as constituents of the plant essential oils [26,27]. Additionally, among the polar fraction metabolites, none has been reported in all the compositions present in literature. Yet, diterpenes and biflavonoids represent the most common compounds in this context. By the way, these compounds have actually been used as chemotaxonomic markers at the genus level even if their occurrence seems, now, to be extended at the whole family level [22,28,29,30,31,32,33]. Several other sub-classes of natural compounds, including triterpenes, lignans, simple flavonoids, and organic acids, have been recorded for this genus [44,45,46,47,50,51,54,55,57,58,62,68,69,71,73,75,77,80,82,84,88]. For what concerns the methodology, in only a few cases, the essential oil was obtained through hydrodistillation [81,83,87] whereas in all the other cases, solvent extraction and solvent distillation methods were used. In all the cases, multiple and different GC analyses were used for the separation and identification of the essential oil metabolites [8,63,65,75,81,83,84]. In this context the presence of improbable natural constituents should be underlined [34], such as siloxane and silyl-derivatives, in the case of the stem bark exudate (resin) of *A. cunninghamii* [84] since in that work the methanolic extract was injected in GC-MS without previous derivatization. In many cases, other identifications techniques such as [α]_D_, TLC, IR, and NMR, alone or together, accompanied the GC analyses [8,63,65]. Indeed, for the analysis of the polar fraction metabolites, SE was the only method for the extraction of these compounds, except one case i.e., the branches of *A. columnaris* from India where US was used [75]. Indeed, CC, TLC, LC, MP, and HPLC techniques, together or separated, were the methods for the separation procedure of the compounds and [α]_D_, IR, NMR, and MS, together or separated, were the methods for the identification procedure of the compounds. In one case, GC-MS was the only method used for the separation and identification of these compounds [58] whereas in others, it accompanied the other methods [62,75]. In one case i.e., the leaves of *A. bidwilli* from Egypt, ECD was another method used for the phytochemical study [69]. In one case, a 2D-TLC screening was used as method for the phytochemical screening of the extract [88]. Lastly, in one case i.e., *A. araucana*, nothing about the methodology could be written since it was not accessible [66].

### 3.4. Ethnopharmacology

The ethnopharmacological uses of species belonging to the *Araucaria* genus are quite numerous.

In particular, in Brazil, *A. angustifolia* leaves are used as emollient, antiseptic, and to treat respiratory infections and rheumatisms. Their dyes are also used for the treatment of wounds and herpes eruptions [90]. In addition, the tinctures derived from the nodes are employed to treat rheumatism. The infusions of the nodes are used orally for the treatment of kidney diseases and sexually transmitted diseases. The infusions of the bark are used topically to treat muscular tensions and varicose veins. The syrup produced of the resin is used for the treatment of respiratory infections [91].

*A. araucana* resin has been used by Amerindian Mapuche tribes located in Southern Chile and Argentina to treat contusions, ulcers, as well as to help cicatrization of skin wounds [60,61].

*A. bidwillii* bark is employed in South Africa against amenorrhoea and as a body and steam wash [92]. Moreover, it is employed in the Lahu tribes of Northern Thailand to treat insomnia [93].

*A. cunninghamii* bark is used by the Yali tribe in West Papua for thatching and in several rituals [94].

*A. heterophylla* aerial parts are used in Peru for toothache and to extract teeth [95].

### 3.5. Biological Activities

#### 3.5.1. Extracts

The biological activities associated with the essential oil or the extracts of *Araucaria* species are also quite numerous.

The biflavonoid rich fraction derived from the fresh needles of *A. angustifolia* has shown to be a potent UV-A UV-B radiation protector [53] as well as to protect liposomes against peroxidative degradation caused by UV-irradiation [52]. The ethyl acetate and *n*-butanol fractions derived from the whole plant of *A. angustifolia* showed strong antiviral effects against HSV-1 with IC_50_ values equal to 8.19 and 11.04 μg/mL, respectively [56]. The ethanol and water extracts of its seeds showed good antioxidant properties [55]. The hydroalcoholic and ethyl acetate extracts of its dead bark showed high antioxidant properties in the DPPH assay with IC_50_ values equal to 1 and 0.9 μg/mL, respectively. Moreover, the same extracts showed medium activity in the lipid peroxidation assay induced by UV, ascorbyl, and hydroxyl free radicals with IC_50_ values equal to 36 and 25 μg/mL, respectively, for the former case, 18 and 17 μg/mL, respectively, for the second case and 12 and 22 μg/mL, respectively, for the latter case [46]. Indeed, the water extract of its female strobili also exerts a time-dependent antiproliferative activity against HEp-2 (human laryngeal cancer) cell lines. In particular, at the concentrations of 250 and 500 μg/mL, it was able to inhibit tumor growth by about 50% after 24 h from the subministration whereas, after 48 h, the percentage of inhibition was about 65 and 70%, respectively, and after 72 h, for both, the percentage of inhibition was 80%, approximately [48]. The same extract showed good DPPH and SOD activities with IC_50_ values equal to 10.0 and 14.7 μM, respectively, as well as good antimutagenic effects against H_2_O_2_ in three different loci i.e., *Lys*, *His*, and *Hom* at the concentrations of 0.05, 0.1, and 0.15% with a percentage of survivals of 100% [49]. The water extract of its bracts at the concentration of 50 μg/mL is also able to completely avoid, in human lung fibroblast cells, cell mortality, protein damage, and SOD and CAT depletions induced by H_2_O_2_ [45].

The crude *A. araucana* resin possesses dose-dependent gastroprotective effects on ethanol–HCl-induced gastric lesions in mice [60]. In addition, the methanol extract of its wood showed moderate antibacterial activity against *Citrobacter pilifera*, *Bacillus subtilis*, *Micrococcus luteus*, and *Staphylococcus aureus* with growth inhibition percentages around 20%, which are values much lower than gentamicin used as control. Indeed, the same extract showed moderate antifungal activities against *Mucor miehei*, *Paecilomyces variotii*, *Ceratocystis pilifera*, and *Trametes versicolor* with growth inhibition percentages from 28.7% for the second one to 57.1% for the latter one. The relative IC_50_ values were in the range 1250–2000 μg/mL [62].

The petroleum ether and methanolic extracts of the leaves and oleoresin of *A. bidwillii*, at the doses 300 mg/kg for the former ones and 100 mg/kg for the latter ones, possess strong anti-insomnia, analgesic, and anti-inflammatory activities [96]. In addition to this, the ethanol extract of its leaves, at the dose of 5 mg/Kg, exerts strong anti-inflammatory activity with percentages of inhibition similar to those of indomethacin i.e., 68.51% vs. 63.28%, respectively [97]. The same extract demonstrated high antinociceptive effects at the concentration of 300 mg/Kg in four different tests: the hot plate test, the acetic acid-induced writhing test, the carrageenan-induced edema test, and the serotonin-induced rat paw oedema test. The associated percentages of inhibition were equal to 81.69%, 54.64%, 45.64%, and 40.75%, respectively. All these values are comparable with those reported for the standard compounds in the relative tests [97]. In addition, its methanolic and ethyl acetate extracts derived from the leaves are able to exert good antitumor effects against HL-60 and K-562 (chronic myelogenic leukemia) cancer cell lines with IC_50_ values equal to 33.11 and 39.81 μg/mL, respectively, for the former and 28.18 and 34.64 μg/mL, respectively, for the latter [98]. The leaf methanol extracts at the concentration of 100 μg/mL showed also strong anti-inflammatory activity acting as an inhibitory agent on the levels of IL-1β, TNF-α by reducing them by 58.4% and 56.4%. Indeed, for what concerns IL-6, the effect was observed to be concentration-dependent. Additionally, the *n*-butanol polyphenolic rich extract at the concentration of 10 μg/mL showed these effects but in minor extent i.e., 44.8% inhibition on the levels of IL-1β and 33.6% inhibition on the levels of TNF-α. Indeed, for what concerns IL-6, also this effect was concentration-dependent. All these values were quite similar to those observed for indomethacin [68]. Its oleoresin possesses good antipyretic activity on female albino rats at the dose of 100 mg/Kg showing the maximum decrease in the rectal temperature after 60 min (−1.35 °C) [71].

The ethanol extract of the branches of *A. columnaris* showed good antioxidant and antiradical activities in absolute with values equal to 93.14 and 74.12% for the DRSC (DPPH radical scavenging activity) and NOSC (nitric oxide scavenging capacity) assays, respectively. Moreover, it also showed a good ferric reducing antioxidant power with a value equal to 113.05  mg Fe(II)E/g FS, a good cupric ion reducing capacity with a value equal to 128.34 mg TE/g FS. Indeed, its dichloromethane extract showed good TAC and MCA activities with values equal to 93.26 mg AAE/g FS and 81.50 mg EDTAE/g FS, respectively [75]. The 70% aqueous methanol extract of the needles of *A. columnaris* showed moderate antioxidant effects with a SC_50_ value equal to 73.0 μg/mL which is, anyway, much higher than ascorbic acid (SC_50_ = 8.0 μg/mL) [73]. Different extracts of its leaves showed to possess also medium antioxidant properties and good α-amylase inhibitory and antibacterial effects against *Pseudomonas* and *Klebsiella* spp. and *Escherichia coli* [76]. These results were also confirmed by another study by Zaffar et al. [99]. Indeed, the study performed by Joshi et al. [100] demonstrated that these extracts were also active against *Xanthomonas phaseoli* and *Erwinia chrysanthemi* with the best MIC values i.e., 62.5 μg/mL for the methanol and *n*-hexane extracts for both bacterial strains.

The methanolic extract of *A. columnaris* bark exerts strong antibacterial effects against *Staphylococcus aureus*, *Escherichia coli* and *Bacillus subtillis* with maximum inhibition zones equal to 20, 18 and 15 mm, respectively. The same extract was also found to be quite cytotoxic against HEK (human kidney) cancer cell line, having an IC_50_ value equal to 95.0 μg/mL [74].

The extracts of *A. cunninghamii* leaves in different solvents (*n*-hexane, chloroform, ethanol, methanol) possess good antifungal activities against *Alternaria alternata*, *Colletotrichum falcatum*, *Fusarium oxysporum*, *Pyricularia oryzae*, *Sclerotinia rolfsii*, *Sclerotinia sclerotiorum*, and *Tillatia indica* with inhibition percentages from 39% for the chloroform extract against *A. alternata* to 57% of the *n*-hexane extract against *A. alternata* itself. All the extracts were active except the *n*-hexane and chloroform ones against *Fusarium oxysporum*. Most of the extracts showed percentages of inhibition similar or better than clotrimazol used as reference [101]. The methanolic extract of its leaves also showed good DPPH radical scavenging activities with an IC_50_ value equal to of 181.9 μg/mL as well as a little reducing power (IC_50_ = 1384.42 μg/mL) and a moderate prevention effect of nitric oxide radical (IC_50_ = 1026.51 μg/mL) [82]. Moreover, the extracts of *A. cunninghamii* stem bark resin in different solvents showed different biological activities. In particular, the methanol extract showed high α-glucosidase inhibition effects with a percentage equal to 48.48% which is very close to that of acarbose i.e., 48.69. The *n*-hexane and dichloromethane effects were lower i.e., 24.2% and 26.58%, respectively. The dichloromethane showed strong cytotoxic effects against in Chang liver cells with an IC_50_ value equal to 92.9 μg/mL. The *n*-hexane and methanol extracts showed minor effect with IC_50_ value equal to 386 and above 500 μg/mL, respectively [84]. In addition to this, its essential oil derived from the foliage showed moderate antibacterial activity against *Salmonella typhimurium*, *Staphylococcus aureus*, and *Staphylococcus epidermidis* with inhibition zones equal to 9, 6, and 5 mm, respectively, whereas it was low against *Staphylococcus aureus* and *Bacillus subtilis* with inhibition zones both equal to 4 mm. Indeed, its essential oil derived from the resin was moderately active only against *Staphylococcus aureus* with an inhibition zone equal to 5 mm. The relative MIC values were in the range 250 and 500 μg/mL and the minimum bactericidal concentrations were 1000 μg/mL or more [81].

The resin extract of *A. heterophylla* stems showed strong cytotoxic effects in vitro against colon (HCT116) and breast (MCF7) human cancer cell lines with IC_50_ values equal to 0.54 and 0.94 μg/mL, respectively, which are quite similar to those observed for doxorubicin (0.70 and 0.96 μg/mL, respectively) [86]. The extracts of its leaves in different solvents showed strong to weak anticancer activity against HEPG-2 (hepatocellular carcinoma), MCF-7, PC-3 (human prostate cancer), and Hela (epitheliod carcinoma) cell lines. In particular, the *n*-butanol extract was one of the most effective with IC_50_ values equal to 12.06, 9.13, 17.42, and 7.69 μg/mL, respectively. These values are lower than doxorubicin but absolutely comparable. The ethyl acetate extract was the most efficient one against MCF-7 and Hela cancer cell lines with IC_50_ values equal to 7.64 and 6.72 μg/mL, respectively. The water extract was more effective against Hela cell lines with an IC_50_ value to 9.84 μg/mL. The petroleum ether and dichloromethane extracts showed quite moderate activities against all the studied cancer cell lines with IC_50_ values above 20 μg/mL, except for the petroleum ether extract against Hela whose IC_50_ value was 19.34 μg/mL [85].

#### 3.5.2. Phytochemicals

In literature there are also some works about the biological activities associated with specific compounds isolated from *Araucaria* species.

Protocatechuic acid, quercetin, (–)-epiafzelechin protocatechuate, and (–)-epiafzelechin *p*-hydroxybenzoate extracted from *A. angustifolia* dead bark from Brazil showed all antioxidant effects in the DPPH assay with IC_50_ values ranging from 0.6 μM of quercetin to 11 μM of (–)-epiafzelechin *p*-hydroxybenzoate. Moreover, only quercetin and (–)-epiafzelechin protocatechuate showed activity in the lipid peroxidation assay and only in that induced by UV and ascorbyl free radicals with IC_50_ values equal to 9 and 21 μM, respectively, for the former case and 30 and 35 μM, respectively, for the second case [46].

Imbricatolic acid, 15-hydroxy-imbricatolal, and 15-acetoxy-imbricatolic acid isolated from *A. araucana* resin from Chile showed dose-dependent gastroprotective effects on ethanol-HCl-induced gastric lesions in mice with maximum activity at doses up to 100 mg/Kg [60]. Moreover, at the dose of 100 mg/kg, 15-hydroxy-imbricatolal, 15-acetoxy-imbricatolic acid, and 15-acetoxylabd-8(17)-en-19-ol were also seen to have effects similar to those of lansoprazole, a standard a proton pump inhibitor drug, reducing the lesions by 78, 69, and 73%, respectively [61]. In addition to this, 15,19-diacetoxylabd-8(17)-en, again isolated from *A. araucana* resin, was observed to possess a good cytotoxic activity against AGS cells (human gastric epithelial) and fibroblasts after treatment for 24 h with IC_50_ values equal to 52 and 72 μM, respectively. These values are much better than those observed for lansoprazole (IC_50_ values equal to 162 and 306 μM, respectively) [61]. Additionally, *seco*-isolariciresinol, pinoresinol, eudesmin, lariciresinol extracted from the wood of this species from Chile showed weak antibacterial activities against *Citrobacter pilifera*, *Bacillus subtilis*, *Micrococcus luteus*, and *Staphylococcus aureus* with growth inhibition percentages below 20%, which are, again, values much lower than gentamicin used as control. Indeed, the same compounds showed moderate antifungal activities against *Mucor miehei*, *Paecilomyces variotii*, *Ceratocystis pilifera*, and *Trametes versicolor* with growth inhibition percentages above 20.0% but below 50%. Actually, pinoresinol was the only compound not active against *Paecilomyces variotii* whereas the *seco*-isolariciresinol was the most active in all the cases with growth inhibition percentages equal to 29.7%, 21.9%, 41.5%, and 45.1%, respectively [62].

The two compounds extracted from the *A. bidwillii* leaves from Egypt, 7-hydroxy-labda-8(17),13(16),14-trien-19-yl-(*E*)-coumarate and 7-hydroxy-labda-8(17),13(16),14-trien-19-yl-7′-*O*-methyl-(*Z*)-coumarate, showed potent cytotoxic effects against L5178Y (mouse lymphoma) cancer cell line with IC_50_ values equal to 2.22 and 1.42 μM, respectively. These values revealed a major effectiveness of these two diterpenoids than the standard drug, kahalalide F, which has an IC_50_ value equal to 4.30 μM [69].

The biflavonoid rich fraction from *A. bidwillii*, both at the concentration of 100 and 200 mg/Kg, was also observed to be a strong protective agent against ischemia-induced oxidative stress in rats by inhibiting free radicals generation, by scavenging reactive oxygen species and by modulating the intracellular antioxidants against ischemia/reperfusion-induced decreases [102].

Ent-19-(*Z*)-coumaroyloxy-labda-8(17),13(16),14-triene, and labda-8(14),15(16)-dien-3β-ol extracted from *A. cunninghamii* aerial parts from China exhibited modest inhibitory effects against *E. coli* with MIC values equal to of 31.9 and 36.3 µM, respectively. Moreover, ent-19-(*Z*)-coumaroyloxy-labda-8(17),13(16),14-triene possess moderate antitumor activity against HL-60 and SMMC-7721 (human hepatoma) cancer cell lines with IC_50_ values equal to 8.90 and 11.53µM, respectively [79].

5-*p*-*cis*-coumaroyl-quinic acid isolated from the twigs and leaves of *A. cunninghamii* showed good antifungal activity against *Helminthosporium sativum*, *Rhizoctonia solani* Kuhn, *Fusarium oxysporum* f. sp. *niveum* and *Fusarium oxysporum* f. sp. *cubense* with EC_50_ values equal to 42.3, 90.0, 62.7 and 100.2 μg/mL, respectively. In addition, this same compound and 4′,7,7′’-*O*-trimethyl-cupressuflavone also showed moderate antibacterial activities against *Escherichia coli*, *Bacillus cereus*, *Staphyloccocus aureus*, *Erwinia carotovora*, and *Bacillus subtilis* with MIC values equal in sequence to 62.5, 62.5, 62.5, 7.8, 15.5 μg/mL and 31.3, 62.5, 62.5, 125.0, and 125.0 μg/mL, respectively. Anyway, most of these data are worse than those observed for ampicillin [80].

Labda-8(17),14-diene and 13-*epi*-cupressic acid isolated from the resin extract of *A. heterophylla* stems showed moderate cytotoxic effects in vitro against colon (HCT116) and breast (MCF7) human cancer cell lines with IC_50_ values ranging from 2.33 μg/mL for 13-*epi*-cupressic acid against MCF7 to 8.04 μg/mL for 13-*epi*-cupressic acid against HCT116. Indeed, 13-*O*-acetyl-13-*epi*-cupressic acid was only active against MCF7 with an IC_50_ value equal to 9.77 μg/mL. All these values are much higher than those observed for doxorubicin [86].

### 3.6. Other Facts

In literature, one interesting curiosity about the *Araucaria* species is also reported. In particular, the edible part of the seeds of *A. angustifolia* are eaten by animals and people for their high nutritional value [47].

## 4. Genus *Columbea*

The overall data of this genus including its morphological description, its distribution, its phytochemistry, its ethnopharmacological uses, and its pharmacological activities are associated to the data of its only existing species i.e., *Columbea brasiliensis* (A. Rich.) Carrière. Yet, besides its distribution which is endemic to Brazil [103], there are no data reported in literature for what concerns the other arguments.

## 5. Genus *Wollemia*

### 5.1. Botany

The overall data of this genus including, its morphological description, its distribution, its phytochemistry, its ethnopharmacological uses, and its pharmacological activities are associated to the data of its only existing species i.e., *Wollemia nobilis* W.G.Jones, K.D.Hill and J.M.Allen.

**Figure 3:**
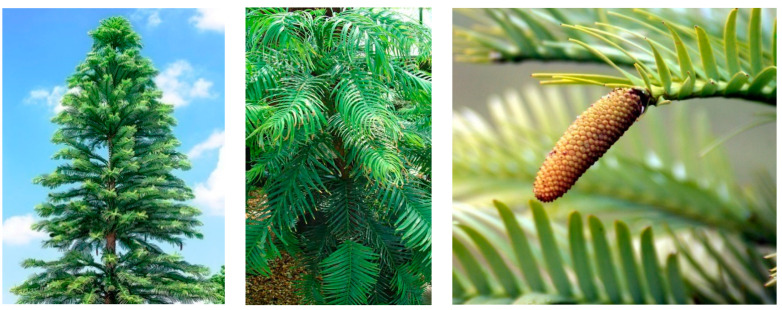
Images of the organs of *Wollemia nobilis*: tree (**left**), leaves (**middle**), cones (**right**).

This species is a monoecious tree which can reach up to 40 m tall. The bark is brown. The stem is multiple with a complex root system. The branching is vertical and lateral. The leaves are flattened, and arranged spirally on the shoots but twisted at the base to form two or four ranks flattened at their own time and they open in November or December depending on the location of the tree. There are two different cones. The male ones (the pollen) are conic and large with a brown-reddish color. Indeed, the female ones (the seeds) are lighter in color and narrower. These cones are disposed in lower positions than the male cones (Figure 3) [104,105].

### 5.2. Distribution

This species is native to Australia and is very rare. In fact, it grows wild only in three different localities within the Wollemi National Park, NSW of Australia where 20 large trees (up to 40 m in height) and 20 juvenile trees are present. Additionally, it was considered to be extinct until 1994. Given these elements, it has been subjected to several protection and conservation programs with the aims to keep its growth habitat secret, monitor them against illegal visits, and develop a plan that favors its cultivation and marketing all around the world. Right because of this, nowadays several *W. nobilis* exemplars are now hosted in a few Botanical Gardens outside Australia (including Italy) as well as in thousands of Australian home gardens. The species prefers sandy soils with good drainage and watering [104,105].

### 5.3. Phytochemistry

Table 3 shows data of all the compounds identified in *W. nobilis*.

As Table 3 clearly shows, most of the phytochemical works reported in the literature about this species regard exemplars collected in Italy [28,29,30,31,32,33]. Yet, this fact is not so unusual since, as already mentioned, Italy is one of the main places where this species has been introduced in order to favor its survival. One only work has regarded the phytochemistry of an exemplar from Australia but only for the essential oil composition [8]. In addition, there are two works regarding the essential oil content of a sample collected in Belgium [106] and one regarding the polar fraction metabolites of an exemplar purchased from a company in Poland [107]. The studied organs were mainly the leaves [8,32,106] but also other organs were taken into considerations i.e., twigs [106,107], half-matured female cones [33], male reproduction organs [29], unripe female cones [31], and male cones for two different studies in the time distance of one year [28,30]. For what concerns the identified compounds, also *W. nobilis* is known to biosynthesize both components of the essential oil and polar fraction metabolites. For none of the studied samples, both essential oil and polar fraction composition were studied. In particular, only the essential oil composition was analyzed for the exemplar from Australia [8] and for the leaves and twigs of the exemplar coming from Belgium [106], whereas only the polar fraction composition was analyzed in all the other cases [28,29,30,31,32,33,107]. Among the essential oil metabolites, only β-pinene and germacrene D are present in all the studied samples [8,106]. Yet, it is still not possible to draw a general conclusion on this matter since the exemplars studied until now are still too few. Anyhow, they cannot be considered as chemotaxonomic markers since they represent very common compounds of the essential oils of different plants. Among the polar fraction metabolites, none have been evidenced in all the studied samples. Yet, biflavonoids and diterpenes have been generally evidenced in all the studied samples [28,29,30,31,32,33,107], but they are mostly considered as chemotaxonomic markers at the family level. For what concerns the methodology, in one case, the essential oil was obtained through solvent distillation [8] whereas in two cases, it was obtained through hydrodistillation [106]. In one case, GLC was also used for the separation of the essential oil metabolites [8] whilst in all the other cases GC-MS was used for the separation and identification of the essential oil metabolites [8,106]. In one case [8], [α]_D_ and NMR analyses accompanied the GC ones. Indeed, for the analysis of the polar fraction metabolites, SE was the only method for the extraction of these compounds [28,29,30,31,32,33,107]. CC was the method for the separation procedure of the compounds in most cases [28,29,30,31,32,33] whereas HPLC and TLC were used in one case [107]. Lastly, NMR and MS techniques were used for the identification procedure of the compounds in all the cases [28,29,30,31,32,33,107]. In one case, UHPLC-HRMS was used for the specific separation and identification of one compound [30].

### 5.4. Ethnopharmacology and Biological Activities

At the moment, in literature, no ethnopharmacological uses and biological activities of the extracts are reported for this species. Nevertheless, it is known that the cones of this species are widely eaten by herbivores [28] and that isocupressic acid and acetyl isocupressic acid are known to exert abortifacient activity in cattle [108] especially in case of late term pregnancy [109].

## 6. Phytochemistry of the Araucariaceae Family

Table 4 shows the phytochemical comparison among all the essential oil metabolites evidenced in the Araucariaceae family.

Table 5 shows, instead, the phytochemical comparison among all the polar fraction metabolites evidenced in the Araucariaceae family.

## 7. Chemotaxonomy of the Araucariaceae Family

As Table 4 and Table 5 clearly show, the phytochemistry of the Araucariaceae family is quite complex. Several metabolites belonging to different classes of natural compounds have been evidenced within it. Yet, none of them have an occurrence spread in all of it even if some compounds have been isolated in many different species. Conversely, other compounds have been isolated in specific species, if not specific exemplars and this fact is not atypical since the qualitative and quantitative content in secondary metabolites of plants is much affected by environmental and genetic factors [26].

**Figure 4:**
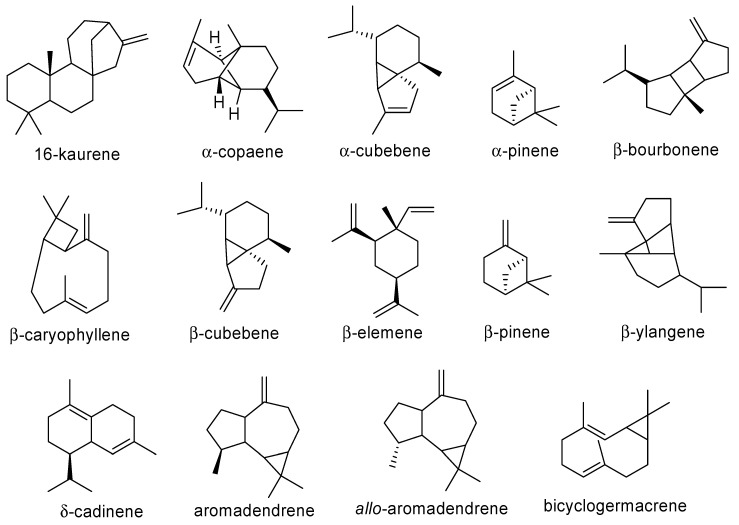
Main essential oil metabolites evidenced in Araucariaceae species—part 1.

**Figure 5:**
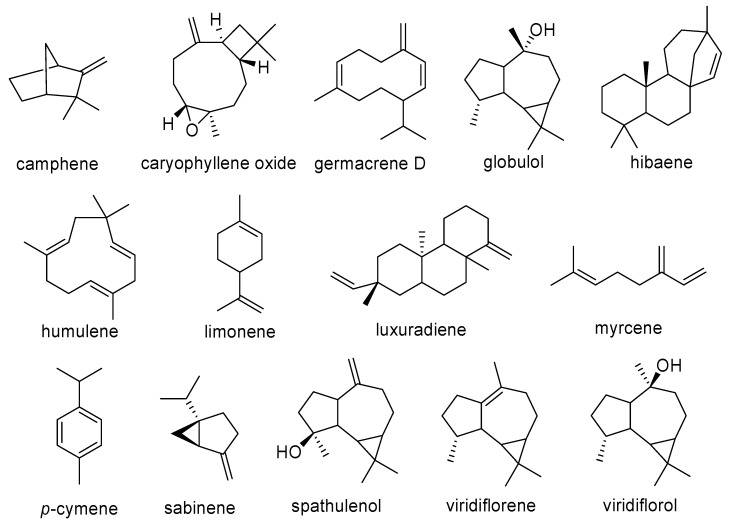
Main essential oil metabolites evidenced in Araucariaceae species—part 2.

Nevertheless, among the essential oil metabolites, the most common compounds were found to be: 16-kaurene, α-copaene, α-cubebene, α-pinene, β-bourbonene, β-caryophyllene, β-cubebene, β-elemene, β-pinene, β-ylangene, δ-cadinene, *allo*-aromadendrene, aromadendrene, bicyclogermacrene, camphene, caryophyllene oxide, germacrene D, globulol, hibaene, humulene, limonene, luxuriadiene, myrcene, *p*-cymene, sabinene, spathulenol, viridiflorene, and viridiflorol (Figure 4 and Figure 5).

Yet, none of these compounds can be actually considered as chemotaxonomic markers since they all are widespread compounds in the plant kingdom [26,27,110,111,112,113,114].

A particular speech regards the presence of alkyl chains. These were evidenced in several species of the family [8] even if not always the exact compounds were identified, but only the general molecular formula. By the way, these compounds are also quite widespread [26,27,110,111,112,113,114].

**Figure 6:**
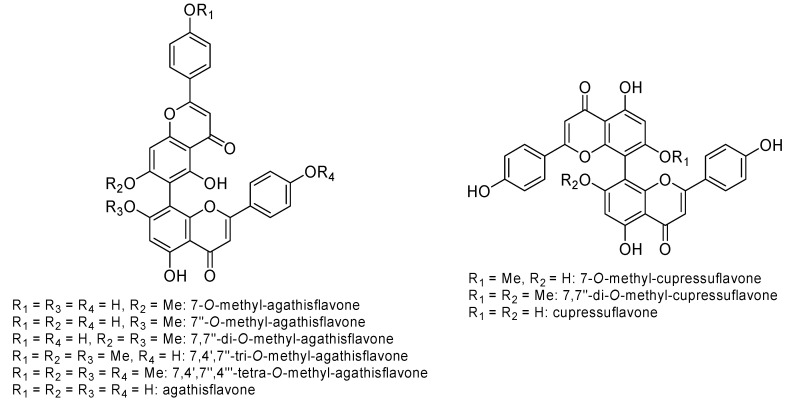
Main polar fractions metabolites evidenced in Araucariaceae species.

Indeed, among the polar fraction metabolites, the most common compounds were found to be: 7-*O*-methyl-agathisflavone, 7-*O*-methyl-cupressuflavone, 7′’-*O*-methyl-agathisflavone, 7,7′’-di-*O*-methyl-agathisflavone, 7,7′’-di-*O*-methyl-cupressuflavone, 7,4′,7′’-tri-*O*-methyl-cupressuflavone, 7,4′,7′’,4′’’-tetra-*O*-methyl-cupressuflavone, agathisflavone, and cupressuflavone (Figure 6).

As previously mentioned, the derivatives of agathisflavone can be really considered as chemotaxonomic markers of the whole Araucariaceae family [22,28,29,30,31,32,33] whereas cupressuflavone and its derivatives have now been started to be evidenced also in other families [115] and so they can no longer be considered as chemotaxonomic markers of the family.

For what concerns the diterpenes, even if no specific compound of this class has been evidenced in all the species reported in literature, as previously mentioned, they are also known to be used as chemotaxonomic markers of the family. In particular, this concerns labdane diterpenes which are, anyway, a very big sub-class of natural compounds and so, they are not so specific also because this kind of compound has been isolated from other families also [116].

## 8. Conclusions

This review article has clearly showed the huge importance of Araucaricaeae species both under different standpoints: phytochemistry, chemotaxonomy, ethnobotany, and pharmacology.

In fact, as for the first point, many compounds, components of the essential oil, and of the polar fraction have been reported in them, including several new ones. For what concerns chemotaxonomy, some compounds could be eventually used as chemotaxonomic markers even if some other studied on this point are necessary in order to develop this concept better. Several species of the Araucariaceae family are also used for ethnobotanical and ethnopharmacological purposes, especially the species belonging to the *Araucaria* genus. Lastly, several extracts derived from Araucariaceae species as well several compounds isolated from them have been found to possess interesting and amazing pharmacological activities, ranging from the mere antioxidant to the greater cytotoxic effects.

Nevertheless, the studies in these fields about this family are quite limited in several senses, not to say absent for the genus *Columbea*, and this review article wants to be a first of its kind but also an incentive to continue the phytochemical, chemotaxonomic, ethnobotanical, and pharmacological studies on the Araucariaceae family in a specific as well as generic manner.

## Figures and Tables

**Table 1:** Compounds evidenced in *Agathis* species.

Species	Collection Site	Organs Studied	Compound	Study Methods	References
*A. alba* (Lam.) Foxw.	Taiwan	Leaves	agathisflavone, 7′’-*O*-methyl-agathisflavone, 7,7′’-di-*O*-methyl-agathisflvone, 7,4′’’-di-*O*-methyl-agathisflavone, 7-*O*-methyl-cupressuflavone, 7,7′’-di-*O*-methyl-cupressuflavone, bilobetin, **plus other biflavonoids not characterized**	SE, CC, α_D_, IR, UV, NMR	[6]
	n.r.		7-*O*-methyl-agathisflavone, 7,4′’’-di-*O*-methyl-agathisflavone, 7-*O*-methyl-cupressuflavone, 7,7′’-di-*O*-methyl-cupressuflavone	SE, CC, TLC, MP, NMR, MS	[7]
*A. atropurpurea* Hyland	Australia	Leaves	α-pinene, α-fenchene, camphene, β-pinene, sabinene, myrcene, α-terpinene, limonene, β-phellandrene, γ-terpinene, terpinolene, α-cubebene, bicycloelemene, α-copaene, α-gurjunene, β-cubebene, β-ylangene, β-caryophyllene, germacrene D, δ-cadinene, bicyclogermacrene, *epi*-cubenol, globulol, viridiflorol, spathulenol, alkyl chain (C_20_H_32_), luxuriadiene, phyllocladene, 16-kaurene	SD, GLC, GC-MS, [α]_D_, NMR	[8]
	New Zealand		agathisflavone, 7′’-*O*-methyl-agathisflavone, 7-*O*-methyl-cupressuflavone, 7,7′’-di-*O*-methyl-agathisflavone, 7,7′’-di-*O*-methyl-cupressuflavone, 7,4′,7′’-tri-*O*-methyl-agathisflavone, 7,4′,7′’-tri-*O*-methyl-cupressuflavone	SE, CC, TLC, LC, NMR, MS	[9]
	Australia	Resin	tricyclene, camphene, dehydro-1,8-cineol, limonene, γ-terpinene, terpinolene, *m*-cymenene, 1,3,8-*p*-menthatriene, 1-octen-3-yl acetate, 1,5,8-*p*-menthatriene, *cis*-β-terpineol, α-terpineol, dihydro-carveol, carvone, bornyl acetate, 2,5-dimethoxy-*p*-cymene, β-bisabolene	HD, GC-MS	[10]
*A. australis* (D. Don) Lindl.	Australia	Leaves	tryciclene, α-pinene, camphene, limonene, α-cubebene, bicycloelemene, α-copaene, β-bourbonene, α-gurjunene, β-cubebene, β-ylangene, β-copaene, β-caryophyllene, aromadendrene, *allo*-aromadendrene, humulene, alkyl chains (C_15_H_24_), viridiflorene, germacrene D, δ-cadinene, bicyclogermacrene, calacorene, palustrol, cubenol, *epi*-cubenol, globulol, viridiflorol, alkyl chain (C_15_H_24_O), spathulenol, T-cadinol, T-muurolol, α-cadinol, hibaene, alkyl chain (C_20_H_32_), sclarene, luxuriadiene, 16-kaurene, alkyl chain (C_20_H_34_O)	SD, GLC, GC-MS, [α]_D_, NMR	[8]
	New Zealand		agathisflavone, 7′’-*O*-methyl-agathisflavone, 7-*O*-methyl-cupressuflavone, 7,7′’-di-*O*-methyl-agathisflavone, 7,7′’-di-*O*-methyl-cupressuflavone, 7,4′,7′’-tri-*O*-methyl-agathisflavone, 7,4′,7′’-tri-*O*-methyl-cupressuflavone, 7,4′,7′’,4′’,-tetra-*O*-methyl-agathisflavone, 7,4′, 7′’,4′’-tetra-*O*-methyl-cupressuflavone	SE, CC, TLC, LC, NMR, MS	[9]
*A. borneensis* Warb.	Malaysia	Leaves	1,2,4a,5,6,8a-hexahydro-1-isopropyl-4,7-dimethyl-naphthalene, 1,4-pentadien-3-ol, 1-iodo-2-methylundecane, 2(1*H*)-phenanthrenone, 2,4,6-trimethyl-octane, abietate, androstenone, bicetyl, 4-methylene-2,8,8-trimethyl-2-vinyl-bicyclo [5.2.0]nonane, caryophyllene oxide, copaene, dodecane, farnesane, heptacosane, methyl-isobutyrate, naphthalene, *n*-heptadecane, *n*-hexacosane, *n*-octacosane, *nor*-pristane, *n*-pentacosane, *n*-pentadecane, *n*-pentadecanoic acid, *n*-tetradecane, octane, 2,3,3-trimethyl-octane, octyl ether, palmitic acid, *trans*-phytol, α-cubenene, β-caryophyllene	SE, GC-FID, GC-MS	[11]
		Stem bark	farnesol, 1,2,4a,5,6,8a-hexahydro-1-isopropyl-4,7-dimethyl-naphthalene, 1,5,9,9-tetramethyl-1,4,7-cycloundecatriene, 4,4,5-trimethyl-2-hexene,3-ethyl-2,7-dimethyl octane, 6-dimethylcyclohexene, 8a(2*H*)-phenanthrenol, 8-methylene, bicetyl, 4,11,11-trimethyl-8-methylene-bicyclo[7.2.0]undec-4-(*Z*)-ene, cetane, copaene, cyclohexene, eicosane, germacrene D, heptacosane, 3-ethyl-3-methyl-heptane, icosane, naphthalene, 1,2,3,4,4a,5,6,8a-octahydro-7-methyl-4-methylene-1-(1-methylethyl)-(1α,4aα)-naphthalene, *n*-docosane, *n*-heptadecane, *n*-hexacosane, *n*-nonadecane, *n*-octacosane, *nor*-pristane, *n*-pentacosane, *n*-pentadecane, *n*-tetratriacontane, *n*-triacontane, octadecane, octadecyl iodide, sorbaldehyde, thiophene, untriacontane, α-caryophyllene, α-cubenene, β-caryophyllene, β-cubebene, methyl-β-D-mannofuranoside, δ-cadinene		
*A. dammara* (Lamb.) Rich. and A.Rich.	China	Leaves	α-tricyclene, α-pinene, camphene, sabinene, β-pinene, β-myrcene, α-phellandrene, (+)-4-carene, *o*-cymene, limonene, γ-terpinene,terpinolene,terpinen-4-ol, α-terpineol, α-copaene, germacrene D, β-bisabolene, δ-cadinene, α-bisabolol	HD, GC-FID, GC-MS	[12]
	Philippines		alkaloids, anthraquinones, tannins, flavonoids, saponins, phenolics (**exact compounds not specified**)	Phytochemical screening	[13]
*A. lanceolata* Warb.	New Caledonia	Resin	19-noranticopalic acid, agatholic acid, sandaracopimaradienol, methyl-sandaracopimarate	SE, CC, [α]_D_, IR, NMR, MS	[14,15]
*A. macrophylla* (Lindl.) Mast.	Australia	Leaves	α-pinene, myrcene, limonene, *p*-cymene, α-cubebene, bicycloelemene, α-copaene, β-bourbonene, β-cubebene, β-ylangene, β-elemene, β-caryophyllene, aromadendrene, humulene, alkyl chains (C_15_H_24_), germacrene D, α-muurolene, δ-cadinene, calamenene, *p*-cymen-8-ol, calacorene, palustrol, cariophyllene oxide, *epi*-cubenol, spathulenol, 5,15-rosadiene, luxuriadiene,16-kaurene	SD, GLC, GC-MS, [α]_D_, NMR	[8]
	China	Aerial parts	(4*S*,5*R*,9*S*,10*R*)-methyl-19-hydroxy-15,16-dinorlabda-8(17),11-*E*-dien-13-oxo-18-oate, (4*R*,5*R*,9*R*,10*R*,13*S*)-13-hydroxypodocarp-8(14)-en-19-oic acid, (4*R*,5*R*,9*R*,10*R*,13*R*)-13-hydroxypodocarp-8(14)-en-19-oic acid, 15-*nor*-14-oxolabda-8(17),12*E*-dien-19-oicacid, 13-oxo-podocarp-8(14)-en-19-oic acid, 13-oxo-podocarp-8(14)-en-19-oate, 16-hydroxy-8(17),13-labdadien-15,16-olid-19-oic acid, 15ξ-hydroxy-pinusolidic acid, lambertianic acid, methyl lambertianate, pinusolidic acid, pinusolide, angustanoic acid F, 8,11,13-abietatrien-15-ol	SE, CC, pTLC, HPLC, [α]_D_, CD, UV, IR, NMR, HRMS, XR	[16]
		Leaves and branches	7α,15α-dihydroxystigmast-4-en-3-one, 3β,22,23-trihydroxystigmast-5-en-7-one, 3β-hydroxystigmast-6-one, β-sitosterol, 4(15)-eudesmene-1β,6α-diol, 3β-hydroxymegastigman-5-en-9-*O*-β-D-glucopyranoside, corchoionoside C, (6*S*,9*R*)-roseoside, 2α,3β-dihydroxyurs-12-en-28-oic acid, 2α,3β,19α-trihydroxyurs-12-en-28-oic acid, 2α,3α,19α-trihydroxyurs-12-en-28-oic acid, 4′,4′’’,7,7′’-tetra-*O*-methyl-agathisflavone, amentoflavone, 4′,4′’’-di-*O*-methyl-upressuflavone, quercetin, catechol	SE, CC, TLC, HPLC, [α]_D_, NMR, HRMS	[17]
	Fiji	Leaves	3α-hydroxy-(13*S*)-l6-*nor*-pimar-7-en-l5-oic acid, (13*S*)-pimar-7-en-3α,15,16-triol, kaur-16-en-3α,l3-diol, kauran-3α,l3,16a-triol, agatharesinol, sitosterol, abietic acid, agathic acid	SE, CC, [α]_D_, IR, NMR, MS	[18]
		Resin	abietic acid, agathic acid	SE, CC, IR, NMR, MS	[19]
*A. microstachya* J.F.Bailey and C.T.White	Australia	Leaves	α-pinene, α-fenchene, camphene, β-pinene, sabinene, myrcene, α-terpinene, limonene, β-phellandrene, γ-terpinene, *p*-cymene, terpinolene, α-cubebene, δ-elemene, bicycloelemene, α-copaene, α-gurjunene, β-cubebene, β-ylangene, β-elemene, β-caryophyllene, aromadendrene, *allo*-aromadendrene, γ-elemene, humulene, viridiflorene, α-terpineol, germacrene D, bicyclogermacrene, α-muurolene, δ-cadinene, calamenene, palustrol, caryophyllene oxide, cubenol, *epi*-cubenol, globulol, viridiflorol, spathulenol, T-cadinol, T-muurolol, α-cadinol, δ-cadinol, alkyl chains (C_20_H_32_), phyllocladene, 16-kaurene	SD, GLC, GC-MS, [α]_D_, NMR	[8]
		Resin	*neo*-abietic acid, *cis*-communic acid, *trans*-communic acid, methyl abietate, methyl sandaracopimarate, agathic acid, methyl-15-hydroxy-abietate, methyl-15-hydroxy-dehydroabietate	SE, CC, [α]_D_, IR, UV, NMR, MS	[20]
*A. moorei* (Lindl.) Mast.	Australia	Leaves	α-pinene, myrcene, limonene, *p*-cymene, α-cubebene, α-copaene, β-bourbonene, α-gurjunene, β-cubebene, β-ylangene, β-caryophyllene, aromadendrene, *allo*-aromadendrene, humulene, viridiflorene, alkyl chains (C_15_H_24_), germacrene D, α-muurolene, δ-cadinene, calamenene, calacorene, caryophyllene oxide, globulol, viridiflorol, spathulenol, T-cadinol, T-muurolol, α-cadinol, δ-cadinol, alkyl chains (C_20_H_32_), 5,15-rosadiene, 16-kaurene	SD, GLC, GC-MS, [α]_D_, NMR	[8]
*A. ovata* (C.Moore ex Vieill.) Warb.	Australia	Leaves	limonene, α-cubebene, α-copaene, β-bourbonene, β-caryophyllene, humulene, viridiflorene, alkyl chains (C_15_H_24_), germacrene D, δ-cadinene, calamenene, alkyl chain (C_15_H_24_O), caryophyllene oxide, spathulenol, hibaene, alkyl chains (C_20_H_32_), sclarene, phyllocladene	SD, GLC, GC-MS, [α]_D_, NMR	[8]
	New Zealand		agathisflavone, 7′’-*O*-methyl-agathisflavone, 7-*O*-methyl-cupressuflavone, 7,7′’-di-*O*-methyl-agathisflavone, 7,7′’-di-*O*-methyl-cupressuflavone, 7,4′,7′’-tri-*O*-methyl-agathisflavone, 7,4′,7′’-tri-*O*-methyl-cupressuflavone, 7,4′,7′’,4′’’-tetra-*O*-methyl-agathisflavone, 7,4′,7′’,4′’’-tetra-*O*-methyl-cupressuflavone	SE, CC, TLC, LC, NMR, MS	[9]
*A. philippinensis* Warb	Philippines	Exudate	tricyclene, α-pinene, α-thujene, α-fenchene, camphene, β-pinene, sabinene, limonene, γ-terpinene, (*E*)-β-ocimene, *p*-cymene, terpinolene, 6-methyl-5-hepten-2-one, fenchone, *cis*-sabinene hydrate, α-copaene, dihydro-α-terpineol, terpinen-4-ol, *trans*-pinocarveol, β-terpineol, neral, α-terpineol, carvone, *trans*-piperitol, *trans*-*p*-mentha-1(7),8-dien-2-ol, *trans*-*p*-mentha-1,8-dien-6-ol, *p*-cymen-8-ol, *cis*-*p*-mentha-1,8-dien-6-ol, *cis*-*p*-mentha-1(7),8-dien-2-ol, limonen-10-ol, **plus one not fully characterized**	HD, GC, GC-MS	[21]
*A. robusta* (C. Moore ex F. Muell.) F.M. Bailey	Australia	Leaves	α-pinene, α-thujene, β-pinene, limonene, 1,8-cineol, *p*-cymene, α-cubebene, α-copaene, β-caryophyllene, aromadendrene, *allo*-aromadendrene, humulene, alkyl chain (C_15_H_24_), viridiflorene, germacrene D, α-muurolene, δ-cadinene, caryophyllene oxide, *p*-cymen-8-ol, globulol, viridiflorol, spathulenol, rimuene	SD, GLC, GC-MS, [α]_D_, NMR	[8]
	New Zealand		agathisflavone, 7′’-*O*-methyl-agathisflavone, 7-*O*-methyl-cupressuflavone, 7,7′’-di-*O*-methyl-agathisflavone, 7,7′’-di-*O*-methyl-cupressuflavone	SE, CC, TLC, LC, NMR, MS	[9]
	Italy		agathisflavone, 7′’-*O*-methyl-agathisflavone, cupressuflavone, rutin, shikimic acid, (2*S*)-1,2-di-*O*-[(9*Z*,12*Z*,15*Z*)-octadeca-9,12,15-trienoyl]-3-*O*-β-D-alactopyranosyl glycerol	SE, CC, NMR, MS	[22]
	United Kingdom		glycosides, tannins, flavonoids, saponins, carbohydrates, fixed oil, mucilage (**exact compounds not specified**)	Phytochemical screening	[23]
	India		α-thujene, α-pinene, camphene, β-pinene, 2-pentyl-furan, α-terpinene, *p*-cymene, limonene, methyl-chavicol, δ-elemene, α-cubebene, α-copaene, β-bourbonene, β-elemene, (*E*)-caryophyllene, (*E*)-α-ionone, *cis*-thujopsene, β-copaene, aromadendrene,(*Z*)-β-farnesene, α-humulene, *allo*-aromadendrene, 9-*epi*-(*E*)-caryophyllene, β-chamigrene, γ-muurolene, β-selinene, α-selinene, α-muurolene, γ-cadinene, *epi*-α-selinene, *trans*-calamenene, occidentalol, germacrene B, α-cedrene epoxide, spathulenol, caryophyllene oxide, β-copaen-4-α-ol, carotol, humulene epoxide II, intermedeol, occidentalol acetate, 10-*nor*-calamenen-10-one, rimuene	HD, GC-FID, GC-MS	[24]
		Resin	α-thujene, α-pinene, camphene, thuja-2,4(10)-diene, sabinene, β-pinene, *p*-cymene, limonene, *p*-mentha-3,8-diene, *p*-cymenene, *trans*-sabinene hydrate, 1,3,8-*p*-menthatriene, α-campholenal, *trans*-sabinol, (*E*)-myroxide, *trans*-β-terpineol, pinocarvone, borneol, terpinen-4-ol, *p*-cymen-8-ol, myrtenol, verbenone, *trans*-carveol, carvone, *trans*-sabinene hydrate acetate, *iso*-bornyl acetate, bornyl acetate, *trans*-pinocarvyl acetate, dihydro-carvyl acetate, *cis*-pinocarvyl acetate, myrtenyl acetate, α-terpinyl acetate, aromadendrene, δ-cadinene	HD, GC-FID, GC-MS	[24]
	United Kingdom (purchased)	Seeds	oleic acid, *cis*-vaccenic acid, linoleic acid, α-linolenic acid, bishomolinoleic acid, bishomo-α-linolenic acid, arachidonic acid, 1,5,8,11,14,17-eicosapentaenoic acid, **plus other not specified**	SE, GLC-MS, HPLC	[25]

Legend: [α]_D_ = Specific Rotation; CC = Column Chromatography; CD = Circular Dichroism; GC-FID = Gas Chromatography coupled to Flame Ionization Detector; GC-MS = Gas Chromatography coupled to Mass Spectrometry; GLC = Gas Liquid Chromatography; GLC-MS = Gas Liquid Chromatography coupled to Mass Spectrometry; HD = HydroDistillation; HPLC = High Performance Liquid Chromatography; HRMS = High Resolution Mass Spectrometry; IR = InfraRed Spectroscopy; LC = Liquid Chromatography; MP = Melting Point; MS = Mass Spectrometry; n.r. = not reported; NMR = Nuclear Magnetic Resonance; pTLC = preparative Thin Layer Chromatography; SD = Solvent Distillation; SE = Solvent Extraction; TLC = Thin Layer Chromatography; UV = UltaViolet Spectroscopy; XR = X-ray Spectroscopy.

**Table 2:** Compounds evidenced in *Araucaria* species.

Species	Collection Site	Organs Studied	Compound	Study Methods	References
*A. angustifolia* (Bertol.) Kuntze	Australia	Leaves	α-pinene, β-pinene, myrcene, limonene, *p*-cymene, bicycloelemene, α-copaene, β-bourbonene, β-copaene, β-elemene, β-caryophyllene, aromadendrene, *allo*-aromadendrene, humulene, alkyl chains, viridiflorene, germacrene D, bicyclogermacrene, δ-cadinene, palustrol, globulol, viridiflorol, spathulenol, α-cadinol, hibaene, 15-kaurene, phyllocladene	SD, GLC, GC-MS, [α]_D_, NMR	[8]
	Brazil	Bark	β-sitosterol, eudesmin, sugiol, agathic acid, agatholic acid, imbricatolic acid	SE, CC, [α]_D_, MP, IR, NMR, MS	[44]
		Bracts	catechin, *epi*-catechin, apigenin, quercetin	SE, HPLC-DAD	[45]
		Dead bark	(−)-*epi*-afzelechin protocatechuate, (−)-*epi*-afzelechin *p*-hydroxybenzoate, quercetin, (−)-*epi*-catechin, benzoic acid, *p*-hydroxybenzoic acid, protocatechuic acid	SE, CC, LC, [α]_D_, NMR, MS	[46]
		Cooked seeds	phenolics (**exact compounds not specified**), glucose, fructose, sucrose	Phytochemical screening	[47]
		Female strobili	dodecanoic acid, hexadecanoic acid, 1,3,4,5-tetrahydroxy-cyclohexane-carboxylic acid, 3-*O*-methyl-D-chiroinositol, 4-nitrophenyl-β-D-glucopyranoside, 4′-methoxy-tectorigenin 3-glucoside-dihydro-quercetin, 7,4′,7′’,4′-tetra-*O*-methyl-amentoflavone	SE, MS^n^	[48]
			catechin, *epi*-catechin, rutin	SE, HPLC-UV	[49]
		Knots	eudesmin, *seco*-isolariciresinol, lariciresinol, isolariciresinol, isolariciresinol-4′-methyl ether	SE, CC, [α]_D_, MP, NMR	[50,51]
		Needles	amentoflavone, ginkgetin, **plus other not specified**	SE, HPLC-MS	[52,53]
		Resin	pinoresinol, pinoresinol monomethyl ether, eudesmin, hinokiresinol, isolariciresinol, (−)*seco*-isolariciresinol	SE, CC, TLC, MP, [α]_D_, IR, UV, NMR, MS	[54]
		Seeds	prodelphinidin B, protocatechuic acid, ferulic acid hexoside, catechin,(−)-*epi*-catechin, eriodictyol-*O*-hexoside, quercetin-3-*O*-glucoside, **plus other not specified**	SE, HPLC-DAD-MS	[55]
		Whole plant	bilobetin, 7′’-*O*-methyl-robustaflavone, cupressuflavone	SE, TLC, CC, UV, HPLC, NMR	[56]
		Whole plant **(Cells)**	octadecyl-(*E*)-*p*-coumarate, octadecyl-(*Z*)-*p*-coumarate, octadecyl-(*E*)-ferulate, octadecyl-(*Z*)-ferulate, 7,4′,7′’-tri-*O*-methyl-amentoflavone, 7,4′,4′’’-tri-*O*-methyl-amentoflavone, 4′,4′’’-di-*O*-methyl-amentoflavone, cabreuvin, irisolidone, pinoresinol, eudesmin, lariciresinol, *trans*-communic acid	SE, CC, pTLC, IR, NMR, MS	[57]
	Chile	Resin	*seco*-isolariciresinol, *seco*-isolariciresinol-4-methyl ether-9′-acetate, *seco*-isolariciresinol-9′-acetate, *seco*-isolariciresinol-4-methyl ether-9,9′-diacetate, *seco*-isolariciresinol-9,9′-diacetate, shonanin, lariciresinol, lariciresinol-4′-methyl ether, lariciresinol-4-methyl ether, lariciresinol-4,4′-dimethyl ether-9-acetate, lariciresinol-4-methyl ether-9-acetate, lariciresinol-9-acetate, 5-methoxy-lariciresinol-9-acetate, 5′-methoxy-lariciresinol-9-acetate, 7′-hydroxy-lariciresinol, 7′-methoxy-lariciresinol, 7′-methoxy-lariciresinol-9-acetate, 7′-hydroxy-lariciresinol-9-acetate, pinoresinol, *epi*-pinoresinol, eudesmin, pinoresinol monomethyl ether, 5-methoxy-eudesmin, 5-methoxy-pinoresinol, isolariciresinol, isolariciresinol-acetate, hinokiresinol, nyasol, 4-hydroxy-benzaldehyde, hydroquinone, *p*-coumaric acid, ferruginol	SE, GC-MS	[58]
*A. araucana* (Molina) K.Koch	Chile	Resin	imbricatolic acid, imbricatadiol,15-hydroxy-imbricatolal, 15-hydroxy-imbrcatolic acid, 15-acetoxy-imbricatolic acid, 15-acetoxy-imbricatolal, 15-formiloxy-imbricatolal, 15-acetoxylabd-8(17)-en-19-ol, 15,19-diacetoxylabd-8(17)-en, labd-8(17)-en-15,19-dial, 19-hydroxylabd-8(17)-en-15-oic acid, junicedric acid, sandaracopimaric acid, agatholic acid	SE, CC, TLC, [α]_D_, NMR, MS	[59,60,61]
		Wood	*seco*-isolariciresinol, pinoresinol, eudesmin, lariciresinol, lariciresinol-4-methyl ether	SE, CC, TLC, HPLC, GLC, GC-MS, MP, NMR	[62]
	Germany	Leaves	(−)-α-copaene, (−)-16-kaurene, (−)-δ-cadinene, (+)-15-beyerene, (−)-β-caryophyllene, (−)-trachylobane, (−)-16-atisirene, (−)-rosa-5,15-diene, (−)-13-*epi*-manoyl-oxide, (−)-sclarene	SD, GC, GC-MS, TLC, [α]_D_, NMR	[63]
	India		7-*O*-methyl-agathisflavone, 7”-*O*-methyl-amentoflavone, 7,7”-di-*O*-methyl-cupressoflavone	SE, MP, TLC, NMR	[64]
	New Zealand	Branches	geraniolene, limonene, γ-cadinene, (−)-α-cadinol, hibaene, (−)-trachylobane, (−)-kaurene, (−)-atisirene	SE, GLC, TLC, [α]_D_, IR, NMR, MS	[65]
	n.a.	n.a.	geraniolone, limonene, (−)-trachylobane, (−)-kaurene, (−)-atisirene, hibaene, (−)-*iso*-kaurene, (−)-*iso*-atisirene	n.a.	[66]
*A. bidwilli* Hook.	Australia	Leaves	α-pinene, β-pinene, myrcene, limonene, *p*-cymene, α-cubebene, α-copaene, β-caryophyllene, aromadendrene, *allo*-aromadendrene, humulene, viridiflorene, germacrene D, bicyclogermacrene, δ-cadinene, caryophyllene oxide, cubenol, globulol, viridiflorol, spathulenol, hibaene, 16-kaurene, alkyl chains (C_20_H_32_)	SD, GLC, GC-MS, [α]_D_, NMR	[8]
		Resin	labda-8(20),13-dien-15-oic acid, labda-8(20), 13-dien-15,19-dioic acid, kolavenic acid	SE, GC-MS	[67]
	Egypt	Leaves	4′,7′’-di-*O*-methyl-agathisflavone,7-*O*-methyl-6-hydroxy-apigenin, 4′,4′’-di-*O*-methyl-amentoflavone	SE, HPLC-UV	[68]
			7-hydroxy-labda-8(17),13(16),14-trien-19-yl-(*E*)-coumarate, 7-hydroxy-labda-8(17),13(16),14-trien-19-yl-(*Z*)-coumarate, 7-hydroxy-labda-8(17),13(16),14-trien-19-yl-7′-*O*-methyl-(*E*)-coumarate, 7-hydroxy-labda-8(17),13(16),14-trien-19-yl-7′-*O*-methyl-(*Z*)-coumarate, 7-oxocallitrisic acid, 2-*O*-acetyl-11-keto-boswellic acid, β-sitosterol-3-*O*-glucopyranoside, phloretic acid, 7,4′,7′’-tri-*O*-methyl-agathisflavone, 7,4′,7′’-tri-*O*-methyl-cupressuflavone	SE, CC, HPLC, [α]_D_, UV, ECD, NMR, HR-MS	[69]
	Germany	Leaves	α-cubene, (−)-16-kaurene, β-cubebene, *Z*-biformene, *E*-biformene, sclarene, α-copaene, germacrene D, (−)-7,13-abietadine, δ-cadinene	SD, GC, GC-MS, TLC, [α]_D_, NMR	[63]
	India	Leaves	agathisflavone, cupressuflavone, amentoflavone, 7-*O*-mcthyl-agathistlavone, bilobetin, hinokiflavone, 7,7′’-di-*O*-methyl-agathisflavone, 7,7′’-di-*O*-mcthyl-cupressuflavone, **plus other biflavonoids not characterized**	SE, CC, [α]_D_, IR, UV, NMR	[6]
			agathisflavone, amentoflavone, cupressuflavone, 7-*O*-methyl-agathisflavone, bilobetin, 7-*O*-methyl-cupressuflavone, hinokiflavone, 7,7′’-di-*O*-methyl-agathisflavone, 7,7′’-di-*O*-methyl-cupressuflavone, **plus other biflavonoids not characterized**	SE, CC, TLC, IR, UV, NMR, MS	[70]
		Oleoresin	diterpenes, flavonoids (**exact compounds not specified**)	Phytochemical screening	[71]
	Italy	Oleoresin	methyl *ent*-8β-hydroxy-labd-*E*-l3-en-15-oate, *ent*-8β,15-labd-*E*-13-ene-diol, methyl *ent*-8α-hydroxy-labd-*E*-l3-en-15-oate, *en*t-l5-acetoxy-labda-8,*E*-13-diene, *ent*-labda-8,*E*-13-dien-15-ol	SE, CC, MP, [α]_D_, IR, NMR, MS	[72]
*A. columnaris* (G.Forst.) Hook.	Australia	Leaves	β-pinene, 1,8-cineol,α-cubebene, bicycloelemene, α-copaene, β-bourbonene, β-cubebene, β-ylangene, β-elemene, β-caryophyllene, aromadendrene, *allo*-aromadendrene, humulene, viridiflorene, germacrene D, bicyclogermacrene, α-muurolene, δ-cadinene, calamenene, calacorene, palustrol, cariophyllene oxide, cubenol, *epi*-cubenol, globulol, viridiflorol, spathulenol, T-cadinol, hibaene, sclarene, luxuriadiene, 16-kaurene, alkyl chains	SD, GLC, GC-MS, [α]_D_, NMR	[8]
	Egypt	Needles	taxifolin, taxifolin-3-*O*-glucopyranoside, orientin, vitexin, *iso*-orientin, *iso*-vitexin, gallic acid	SE, CC, TLC HPLC, UV, NMR, MS	[73]
	India	Bark	benzoic acid, 1*H*-*N*-hydrxynaphth(2,3)imidazole-6,7-dicarboximide, 3-4-methoxyphenyl-2-propenoic acid, 4-[[[[(1,2-dichloroethylidene) amino]oxy]carbonyl]amino]-methyl ester benzoic acid, *tert*-butoxy 2 ethoxyethane, *1H*-*N*-hydroxynaphth(2,3-d)imidazole-6,7-dicarboximide, 6-methoxy-2-methyl-2-phenyl-2H-1-benzopyran, 2,3-di-amino-2-methylpropanoic acid, 2,4-dimethyl-furan	SE, TLC, GC-MS	[74]
		Branches	myricetin, catechin, rutin, quercetin, luteolin, chlorogenic acid, ferulic acid, gallic acid, vanillic acid	US, HPLC-MS	[75]
			manool, *N*,*N*-*bis*(2-hydroxyethyl)dodecanamide, palmitic acid, cariophyllene, 1,7,7-trimethyl-3-phenethylidenebicyclo[2.2.1]heptan-2-one, 9-octadecenoic acid, cedr-8-en-15-ol, kaur-16-en-19-ol, 1,3-*bis*-(2-cyclopropyl,2-methylcyclopropyl)-but-2-en-1-one, methyl-(*Z*)-5,11,14,17-eicosatetraenoate, methyl-communate, abietic acid, agathic acid dimethyl ester, docosyl acetate, stigmastan-3,5-diene, 1-heptacosanol, tricosyl acetate, β-sitosterol, β-sitosterol acetate	SE, GC-MS	[75]
		Leaves	saponins, tannins, phenols, flavonoids phytosteroids (**exact compounds not specified**)	Phytochemical screening	[76]
			agathisflavone, amentoflavone, cupressuflavone, 7-*O*-methyl-agathisflavone, hinokiflavone, 7′’-*O*-methyl-amentoflavone, 7,4′’’-di-*O*-methyl-agathisflavone,7,7′’-di-*O*-methyl-agathisflavone, 7,7′’-di-*O*-methyl-amentoflavone, 7,7′’,4′’’-tri-*O*-methyl-agathisflavone, 7,4′,7′’-tri-*O*-methyl-amentoflavone, 7,4′,7′’,4′’’-tetra-*O*-methyl-amentoflavone, 7,4′,7′’,4′’’-tetra-*O*-methyl-cupressuflavone, **plus other biflavonoids not characterized**	SE, CC, TLC, IR, UV, NMR, MS	[70]
		Whole plant	saponins, antraquinones, terpenes, flavonoids, carbohydrates, proteins (**exact compounds not specified**)	Phytochemical screening	[77]
	Pakistan **(purchased from ornamental shop)**	Aerial parts	tannins, flavonoids (**exact compounds not specified**)	Phytochemical screening	[78]
*A. cunninghamii* Mudie	Australia	Leaves	α-pinene, β-pinene, sabinene, myrcene, limonene, 1,8-cineol, *p*-cymene, α-copaene, β-ylangene, β-elemene, β-caryophyllene, aromadendrene, *allo*-aromadendrene, humulene, α-terpineol, germacrene D, bicyclogermacrene, δ-cadinene, *p*-cymen-8-ol, calacorene, palustrol, cariophyllene oxide, globulol, viridiflorol, spathulenol, hibaene, 15-kaurene, 16-kaurene, alkyl chains	SD, GLC, GC-MS, [α]_D_, NMR	[8]
	China	Aerial parts	*ent*-19-(*Z*)-coumaroyloxy-labda-8(17),13(16),14-triene, *ent*-19-(*E*)-coumaroyloxy-labda-8(17),13(16),14-triene, shikimic acid *n*-butyl ester, 5-(*E*)-coumaroyloxy-quinic acid *n*-butyl ester, 5-(*Z*)-coumaroyloxyquinic acid *n*-butyl ester, labda-8(14),15(16)-dien-3β-ol	SE, CC, [α]_D_, IR, UV, NMR, MS	[79]
	China	Twigs and leaves	4-*n*-butoxylphenylpropanetriol, 5-*p*-*cis*-coumaroyl-quinic acid, 5-*p*-*trans*-coumaroyl-quinic acid, quinic acid, (6*R*,9*S*)-3-oxo-α-ionol-9-*O*-β-D-glucopyranoside, (6*S*,9*S*)-roseoside, 5,5′’-dihydroxy-7,4′,7′’,4′’’-tetra-*O*-methyl-biflavone, 7,4′,7′’-tri-*O*-methyl-cupressuflavone	SE, CC, TLC, LC, [α]_D_, IR, UV, NMR, HRMS	[80]
	India	Fresh foliage	*n*-nonane, tricyclene, α-thujene, α-pinene, α-fenchene, sabinene, β-pinene, α-phellandrene, α-terpinene, *p*-cymene, limonene, (*Z*)-β-ocimene, (*E*)-β-ocimene, γ-terpinene, terpinolene, *n*-undecane, terpinen-4-ol, myrtenol, *n*-tridecane, α-copaene, β-panasinsene, (*E*)-caryophyllene, β-copaene, aromadendrene, α-humulene, (*E*)-β-farnesene, *allo*-aromadendrene, germacrene D, α-amorphene, β-selinene, bicyclogermacrene, occidentalol, longipinanol, spathulenol, caryophyllene oxide, humulene epoxide II, α-muurolol, occidentalol acetate, *cis*-thujopsenal, *epi*-cyclocolorenone, hibaene, pimaradiene, dolabradiene, 15-kaurene, luxuriadiene, phyllocladene, 16-kaurene, abietatriene, laurenan-2-one, dehydro-abeitol, **diterpenes**	HD, GC-FID, GC-MS	[81]
		Senescent foliage	*n*-nonane, tricyclene, α-thujene, α-pinene, α-fenchene, camphene, sabinene, β-pinene, α-phellandrene, δ-3-carene, α-terpinene, *p*-cymene, limonene, 1,8-cineol, (*Z*)-β-ocimene, (*E*)-β-ocimene, γ-terpinene, acetophenone, terpinolene, *n*-undecane, 3-octanol acetate, terpinen-4-ol, hexyl butanoate, myrtenol, (3*Z*)-hexenyl-2-methyl butanoate, hexyl *iso*-valerate, 1-tridecene, *n*-tridecane, α-copaene, (3*Z*)-hexenyl hexanoate, sativene, (*E*)-caryophyllene, β-copaene, aromadendrene, α-humulene, (*E*)-β-farnesene, *allo*-aromadendrene, germacrene D, β-selinene, bicyclogermacrene, occidentalol, longipinanol, spathulenol, caryophyllene oxide, viridiflorol, humulene epoxide II, 1,7-di-*ep*i-α-cedrenal, α-muurolol, 14-hydroxy-(*Z*)-caryophyllene, 14-hydroxy-9-*epi*-(*E*)-caryophyllene, occidentalol acetate, *cis*-thujopsenal, *epi*-cyclocolorenone, hibaene, pimaradiene, dolabradiene, 15-kaurene, luxuriadiene, phyllocladene, 16-kaurene, abietatriene, sandaracopimarinol, dehydro-abeitol, **diterpenes**		
		Resin oil	*n*-nonane, α-pinene, sabinene, β-pinene, 6-methyl-5-hepten-2-one, 3-octanol, *p*-cymene, limonene, 1,8-cineol, (*Z*)-β-ocimene, γ-terpinene, terpinolene, *n*-undecane,1-octen-3-yl-acetate, 3-octanol acetate, (3*Z*)-hexenyl-butanoate, hexyl butanoate, myrtenol, (3*Z*)-hexenyl-2-methyl butanoate, hexyl *iso*-valerate, 1-tridecene, *n*-tridecane, α-copaene, (3*Z*)-hexenyl hexanoate, β-panasinsene, sativene,(*E*)-caryophyllene, β-copaene, aromadendrene, α-humulene, (*E*)-β-farnesene, *allo*-aromadendrene, germacrene D, α-amorphene, β-selinene, bicyclogermacrene, occidentalol, longipinanol, spathulenol, caryophyllene oxide, globulol, viridiflorol, humulene epoxide II, 1,7-di-*ep*i-α-cedrenal, α-muurolol, 14-hydroxy-(*Z*)-caryophyllene, 14-hydroxy-9-*epi*-(*E*)-caryophyllene, occidentalol acetate, 5-*neo*-cedranol, *cis*-thujopsenal, cyclocolorenone, squamulosone, *epi*-cyclocolorenone, hibaene, pimaradiene, dolabradiene, 15-kaurene, luxuriadiene, phyllocladene, abietatriene, laurenan-2-one, sandaracopimarinol, dehydro-abeitol, **diterpenes, sesquiterpenoids**		
		Leaves	umbelliferone, quercetin, kaempferol, catechin, *epi*-catechin, chlorogenic acid, gallic acid, caffeic acid, ellagic acid	SE, HPLC	[82]
			7-*O*-methyl-agathisflavone, 7′’-*O*-methyl-amentoflavone, hinokiflavone, 7,4′’’-di-*O*-methyl-agathisflavone, 7,4′-di-*O*-methyl-amentoflavone, 7,7′’-di-*O*-methyl-cupressuflavone, kayaflavone, 7, 4′,7′’-tri-*O*-methyl-cupressuflavone, 7,4′,7′’,4′’’-tetra-*O*-methyl-amentoflavone, 7,4′,7′’,4′’’-tetra-*O*-methyl-cupressuflavone, **plus other biflanoids not characterized**	SE, CC, TLC, IR, UV, NMR, MS	[70]
	Nigeria	Leaves	nonane, β-calacorene, tricyclene, spathulenol, α-pinene, caryophyllene oxide, camphene, campherenone, sabinene, humulene epoxide II, β-pinene, T-muurolol, 2-pentyl-furan, *ar*-turmerone, α-phellandrene, shyobunol, δ-3-carene, beyerene, α-terpinene, kaurene, phyllocladene, (Z)-β-ocimene, laurenene, γ-terpinene, α-thujone, undecane, α-campholenal, δ-terpineol, terpinen-4-ol, menthol, verbenone, *p*-mentha-1,4-dien-7-ol, *trans*-carveol, myrtenol, α-cubebene, α-copaene, β-elemene, β-caryophyllene, aromadendrene, sesquisabinene B, α-humulene, *allo*-aromadendrene, γ-muurolene, germacrene D, β-selinene, bicyclogermacrene, γ-cadinene, δ-cadinene	HD, GC, GC-MS	[83]
	South Africa	Stem bark resin	palmitic acid ethyl ester, (*E*)-9-octadecenoic acid ethyl ester, ethyl heptadecanoate, di-isooctyl adipate, arachidonic acid, cholesterol, *O*-ethyl-hydroxylamine, 3-trimethylsilyloxypropyl hexadecanoate, 9-octadecenoic acid methyl ester, docosahexaenoic acid, *L*-valine-*N*-[*N*-[*N*2,*N*6-*b*i*s*-(1-oxodecyl)-*L*-lysyl]glycyl]-methyl ester, eicosamethyl-cyclodecasiloxane, 1,1-diethoxy-nonane, ethyl 9-octadecenoate, cyclononasiloxane	SE, GC-MS	[84]
			phenolics (**exact compounds not specified**)	Phytochemical screening	
*A. heterophylla* (Salisb.) Franco	Australia	Leaves	α-pinene, camphene, β-pinene, sabinene, myrcene, α-terpinene, limonene, γ-terpinene, *p*-cymene, terpinolene, α-cubebene, α-copaene, β-caryophyllene, aromadendrene, *allo*-aromadendrene, humulene, viridiflorene, germacrene D, bicyclogermacrene, δ-cadinene, cariophyllene oxide, phyllocladene, 16-kaurene, alkyl chains	SD, GLC, GC-MS, [α]_D_, NMR	[8]
	Egypt	Leaves	flavonoids, phenolics (**exact compounds not specified**)	Phytochemical screening	[85]
		Resin from the stems	labda-8(17),14-diene, 13-*epi*-cupressic acid, 13-*O*-acetyl-13-*epi*-cupressic acid	SE, CC, TLC, NMR	[86]
	Germany	Leaves	(−)-α-copaene, (−)-16-kaurene, (−)-germacrene D, sclarene, (−)-β-caryophyllene, 16-phyllocladene, *epi*-zonarene, (−)-sandaracopimaradiene, (−)-8(14),15-pimaradiene, dolabradiene, 9-*epi*-sclarene	SD, GC, GC-MS, TLC, [α]_D_ NMR	[63]
	Hawaii	Leaves	α-pinene, camphene, β-pinene, limonene, α-terpineol, β-caryophyllene	HD, GC-FID, GC-MS	[87]
	India	Foliage	*n*-nonane, α-pinene, camphene, sabinene, *p*-cymene, limonene, γ-terpinene, terpinolene, *n*-undecane, α-copaene, β-bourbonene, (*E*)-caryophyllene, β-copaene, α-humulene, (*E*)-β-farnesene, γ-gurjunene, germacrene D, α-amorphene, viridiflorene, α-muurolene, γ-cadinene, δ-cadinene, spathulenol, globulol, viridiflorol, β-oplopenone, *epi*-α-cadinol, α-muurolol, α-cadinol, cubitene, laurenene, rimuene, *epi*-laurenene, *iso*-pimara-9(11),15-diene, hibaene, *ent*-rosa-5,15-diene, pimaradiene, (3*Z*)-cembrene A, sandaracopimara-8(14),15-diene, dolabradiene, sclarene, 15-kaurene, luxuriadiene, phyllocladene, 16-kaurene, abietatriene, 13-*epi*-manool, abietadiene, phyllocladanol, **diterpenes**	HD, GC-FID, GC-MS	[81]
		Resin oil	*n*-undecane,1-octen-3-yl-acetate, α-cubebene, α-ylangene, α-copaene, β-bourbonene, *iso*-longifolene, β-elemene, (*E*)-caryophyllene, β-copaene, α-*trans*-bergamotene, α-guaiene, α-humulene, β-santalene, *allo*-aromadendrene, γ-gurjunene, germacrene D, aristolochene, *trans*-muurola-4(14),5-diene, α-muurolene, viridiflorene, δ-amorphene, γ-cadinene, δ-cadinene, α-cadinene, α-calacorene, germacrene B, (*E*)-nerolidol, spathulenol, β-oplopenone, *epi*-α-cedrenal, α-muurolol, pimaradiene, sandaracopimara-8(14),15-diene, manool oxide, sandaracopimarinol, **diterpenes**		
		Whole plant	saponins, antraquinones, terpenes, flavonoids, carbohydrates, proteins, (**exact compounds not specified**)	Phytochemical screening	[77]
	Indonesia	Leaves	polyisoprenoids (**exact compounds not specified**)	2D-TLC screening	[88]
*A. hunsteinii* K.Schum.	Australia	Leaves	α-pinene, camphene, β-pinene, sabinene, myrcene, limonene, β-phellandrene, 1,8-cineol, *p*-cymene, terpinolene, α-cubebene, α-copaene, β-bourbebene, β-ylangene, β-elemene, β-caryophyllene, aromadendrene, humulene, viridiflorene, germacrene D, bicyclogermacrene, α-muurolene, calamenene, calacorene, methyl-eugenol, ledol, cubenol, *epi*-cubenol, globulol, viridiflorol, spathulenol, T-cadinol, T-muurolol, sclarene	SD, GLC, GC-MS, [α]_D_, NMR	[8]
*A. luxurians* (Brongn. and Gris) de Laub.	Australia	Leaves	α-pinene, β-pinene, limonene, *p*-cymene, α-cubebene, bicycloelemene, α-copaene, β-caryophyllene, aromadendrene, *allo*-aromadendrene, humulene, viridiflorene, germacrene D, bicyclogermacrene, δ-cadinene, calamenene, palustrol, cariophyllene oxide, cubenol, *epi*-cubenol, globulol, viridiflorol, spathulenol, 5,15-rosadiene, luxuriadiene, 16-kaurene, alkyl chains	SD, GLC, GC-MS, [α]_D_, NMR	[8]
*A. montana* Brongn. and Gris	Australia	Leaves	α-pinene, β-pinene, myrcene, limonene, *p*-cymene, α-cubebene, α-copaene, β-bourbonene, β-caryophyllene, aromadendrene, *allo*-aromadendrene, humulene, viridiflorene, α-terpineol, bicyclogermacrene, α-muurolene, δ-cadinene, cariophyllene oxide, globulol, viridiflorol, spathulenol, 5,15-rosadiene, luxuriadiene, phyllocladene, 16-kaurene, alkyl chains	SD, GLC, GC-MS, [α]_D_, NMR	[8]
*A. muelleri* (Carrière) Brongn. and Gris	Australia	Leaves	myrcene, limonene, *p*-cymene, bycicloelemene, α-copaene, β-ylangene, β-elemene, β-caryophyllene, aromadendrene, *allo*-aromadendrene, humulene, germacrene D, bicyclogermacrene, δ-cadinene, cariophyllene oxide, globulol, viridiflorol, spathulenol, 5,15-rosadiene, sclarene, luxuriadiene, phyllocladene, 15-kaurene, alkyl chain (C_20_H_32_)	SD, GLC, GC-MS, [α]_D_, NMR	[8]
*A. rulei* F.Muell.	India	Leaves	amentoflavone, cupressuflavone, agathisflavone, robustaflavone, 7-*O*-methyl-agathisflavone, 7,4′’’-di-*O*-methyl-agathisflavone, 7,7′’-di-*O*-methyl-cupressuflavone, 7,7′’,4′’’’-tri-*O*-methyl-cupressuflavone, 7,4′,7′’,4′’’-tetra-*O*-methyl-amentoflavone, 7,4′,7′’,4′’’-tetra-*O*-methyl-cupressuflavone	SE, TLC, [α]_D_, MP, NMR	[89]
*A. scopulorum* de Laub.	Australia	Leaves	α-pinene, β-pinene, myrcene, α-cubebene, δ-elemene, α-copaene, β-bourbonene, β-ylangene, β-elemene, β-caryophyllene, aromadendrene, γ-elemene, *allo*-aromadendrene, humulene, viridiflorene, germacrene D, bicyclogermacrene, α-muurolene, δ-cadinene, calamenene, calacorene, cariophyllene oxide, *epi*-globulol, ledol, cubenol, *epi*-cubenol, globulol, viridiflorol, spathulenol, T-cadinol, 5,15-rosadiene, sclarene, luxuriadiene, 16-phyllocladanol, alkyl chains	SD, GLC, GC-MS, [α]_D_, NMR	[8]

Legend: 2D-TLC: Bidimensional Thin Layer Chromatography; [α]_D_ = Specific Rotation; CC = Column Chromatography; CD = Circular Dichrosim; ECD = Electron Capture Dissociation; GC-FID = Gas Chromatography coupled to Flame Ionization Detector; GC-MS = Gas Chromatography coupled to Mass Spectrometry; GLC = Gas Liquid Chromatography; GLC-MS = Gas Liquid Chromatography coupled to Mass Spectrometry; HD = HydroDistillation; HPLC = High Performance Liquid Chromatography; HPLC-DAD: High Performance Liquid Chromatography coupled to a Diode Array Detector; HPLC-DAD-MS = High Performance Liquid Chromatography coupled to a Diode Array Detector and Mass Spectrometry; HPLC-UV = High Performance Liquid Chromatography coupled to a UltraViolet Detector; HRMS = High Resolution Mass Spectrometry; IR = InfraRed Spectroscopy; LC = Liquid Chromatography; MP = Melting Point; MS = Mass Spectrometry; MS^n^ = Tandem Mass Spectrometry; n.a. = datum not accessible; NMR = Nuclear Magnetic Resonance; pTLC = preparative Thin Layer Chromatography; SD = Solvent Distillation; SE = Solvent Extraction; TLC = Thin Layer Chromatography; US = UltraSonication; UV = UltraViolet Spectroscopy; XR = X-ray Spectroscopy.

**Table 3:** Compounds evidenced in *Wollemia nobilis.*

Collection site	Organs Studied	Compounds	Study Methods	References
Australia (Wollemi National Park)	Leaves	α-pinene, camphene, β-pinene, sabinene, myrcene, terpinene, limonene, β-phellandrene, 1,8-cineol, γ-terpinene, *p*-cymene, terpinolene, α-copaene, β-caryophyllene, aromadendrene, *allo*-aromadendrene, humulene, germacrene D, bicyclogermacrene, δ-cadinene, globulol, viridiflorol, spathulenol, hibaene, 15-kaurene, 16-kaurene, alkyl chain (C_15_H_24_)	SD, GLC, GC-MS, [α]_D_, NMR	[8]
Belgium (Arboretum Kalmthou)	Leaves	β-pinene, β-myrcene, 3-methylene-1,7-octadiene, octen-1-ol acetate, 6-camphenol, (*E*)-3(10)-caren-4-ol,verbenol, 6,6-dimethyl-2-methylene-bicyclo[2.2.1]-heptan-3-one, *Z*-β-terpineol, 2-acetyl-2-carene, myrtenol, verbenone, (*E*)-3(10)-caren-2-ol,carvone, 2,2-dimethylvaleroyl chloride, bergamol, (*Z*)-2-decenal, bornyl acetate, *p*-mentha-6,8-dien-2-ol acetate, *p*-menth-8-en-2-ol acetate, 7,11-dimethyl-3-methylene-1,6,10-dodecatriene, cyclobuta[1,2:3,4]dicyclopentenedecahydro-3a-methyl-6-methylene-1-(1-methylethyl)-[1*S*-(1.α,3a.α,3b.β,6a.β,6b.α)], β-cis-ocimene, germacrene D, germacrene B, filipendulal, (*iso*)-aromadendrene epoxide, phylocald-15-ene, kaur-16-ene, [1ar-(1a.α,4.β,4a.β,7.α,7a.β,7b.α]-decahydro-1,1,4,7-tetramethyl-1*H*-cycloprop[e]azulen-4-ol,aromadendrene (2)-oxide, α-cadinol, (*iso*)-geraniol, *trans*-longipinocarveol, *tran*s-*Z*-α-bisabolene epoxide, tetrahydrogeranyl acetate,sandaracopimar-15-en-8.b.-yl-acetate, 4β,17-(acetoxy)-kauran-18-al	HD, GC-MS	[106]
	Twigs	β-pinene, β-myrcene, 3-methylene-1,7-octadiene, δ-carene, octen-1-ol acetate, 6-camphenol, (*E*)-3(10)-caren-4-ol,verbenol, *Z*-β-terpineol, 8-oxo-*cis*-ocimene, myrtenol, 2,2-dimethylvaleroyl chloride, bergamol, bornyl acetate, *p*-mentha-6,8-dien-2-ol acetate, *p*-menth-8-en-2-olacetate, cyclobuta[1,2:3,4]dicyclopentenedecahydro-3a-methyl-6-methylene-1-(1-methylethyl)-[1*S*-(1.α,3a.α,3b.β,6a.β,6b.α)], sativene, 1-ethenyl-1-methyl-2,4-bis(1-methylethenyl) cyclohexane, 7,11-dimethyl-3-methylene-1,6,10-dodecatriene, β-cis-ocimene, germacrene D, germacrene B, α-muurolene, spathulenol, filipendulal, (*iso*)-aromadendrene epoxide, [1*ar*-(1a.α,4.β,4a.β,7.α,7a.β,7b.α]-decahydro-1,1,4,7-tetramethyl-1*H*-cycloprop[e]azulen-4-ol,aromadendrene (2)-oxide, α-cadinol, *trans*-longipinocarveol, caryophyllene oxide, *tran*s-*Z*-α-bisabolene epoxide, tetrahydrogeranyl acetate, sandaracopimar-15-en-8.b.-yl-acetate, phylocald-15-ene, kaur-16-ene, 4β,17-(acetoxy)-kauran-18-al, kaur-16-en-18-oic acid methyl ester		
Italy (Botanical garden of Rome)	Leaves	pheophorbide *a*, isocupressic acid, acetyl-isocupressic acid, sandaracopimaric acid, agathic acid, 7,4′,4′’’-tri-*O*-methyl-agathisflavone, 7,4′,7′’,4′’’-tetra-*O*-methyl-agathisflavone, caffeic acid, shikimic acid	SE, CC, NMR, MS	[32]
	Half-matured female cones	acetyl-isocupressic acid, methyl-(*E*)-communate, sandaracopimaric acid, wollemol, 7′’-*O*-methyl-agathisflavone, 7,4′’’-di-*O*-methyl-agathisflavone, shikimic acid, quinic acid, glucose, sucrose, raffinose, D-lactic acid, succinic acid, alanine	SE, CC, NMR, MS	[33]
	Male reproduction organs	tri-linolenoyl-*sn*-glycerol, 1,2-di-palmitoleoyl-3-myristoyl-*sn*-glycerol, 6′-*O*-acetyl-pina-2-ene-4,10-diol-10-*O*-β-D-glucopyranoside, isocupressic acid, acetyl-isocupressic acid, agathic acid, sandaracopimaric acid, 7,4′,7′’,4′’’-tetra-*O*-methyl-robustaflavone, 7-*O*-methyl-agathisflavone, 7,4′’’-di-*O*-methyl-agathisflavone, 7,4′,4′’’-tri-*O*-methyl-agathisflavone, 7,4′,7′’,4′’’-tetra-*O*-methyl-agathisflavone, 7,7′’,4′’’-tri-*O*-methyl-amentoflavone, shikimic acid, quinic acid, D-lactic acid, glucose, sucrose, pinitol, alanine		[29]
	Unripe female cones	2α-hydroxy-8(14),15-sandaracopimaradien-18-oic acid, wollemolide, 15-formyloxy-imbricatolicacid, 15-formyloxy-imbricatolal, agathisflavone, cupressuflavone, 7′’-*O*-methyl-agathisflavone, 7-*O*-methyl-cupressuflavone, dactylifric acid, shikimic acid, caffeic acid, protocatechuic acid		[31]
	Male cones	wollemolide, isocupressic acid, acetyl-isocupressic acid, methyl (*E*)-communate, sandaracopimaric acid, wollemol, 4′-*O*-methyl-scutellarein, 7-4′’’-dimethoxy-agathisflavone, shikimic acid, glucose, sucrose, arginine	SE, CC, UHPLC-HRMS, NMR, MS	[28,30]
Poland **(purchased from a Company)**	Twigs	7-*O*-methyl-agathisflavone, 7,4′’’-di-*O*-methyl-agathisflavone, 7,7′’-di-*O*-methyl-cupressuflavone, 7,7′’,4′’’-tri-*O*-methyl-agathisflavone, 7,4′,7′’-tri-*O*-methyl-cupressuflavone, 7,4′,7′’,4′’’-tetra-*O*-methyl-cupressuflavone, 7,4′,7′’,4′’’-tetra-*O*-methyl-amentoflavone	SE, HPLC, TLC, NMR, HRMS	[107]

Legend: [α]_D_ = Specific Rotation; CC = Column Chromatography; GC-MS = Gas Chromatography coupled to Mass Spectrometry; GLC = Gas Liquid Chromatography; HD = HydroDistillation; HPLC = High Performance Liquid Chromatography; HRMS = High Resolution Mass Spectrometry; MS = Mass Spectrometry; NMR = Nuclear Magnetic Resonance; SD = Solvent Distillation; SE = Solvent Extraction; TLC = Thin Layer Chromatography; UHPLC-HRMS = Ultra High Performance Liquid Chromatography coupled to High Resolution Mass Spectrometry.

**Table 4:** Occurrence of essential oil metabolites in Araucariaceae species.

Compound	Occurrence in the Family	References
1,1-diethoxy-nonane	*Araucaria cunninghamii*	[84]
1,2,4a,5,6,8a-hexahydro-1- isopropyl-4,7-dimethyl-naphthalene	*Agathis borneensis*	[11]
1,2,3,4,4a,5,6,8aoctahydro-7-methyl-4-methylene-1- (1-methylethyl)-(1α,4aα)-naphthalene	*Agathis borneensis*	[11]
1,3,8-*p*-menthatriene	*Agathis robusta*, *Agathis atropurpurea*	[10,24]
1,3-*bis*-(2-cyclopropyl,2-methylcyclopropyl)-but-2-en-1-one	*Araucaria columnaris*	[75]
1,4-pentadien-3-ol	*Agathis borneensis*	[11]
1,5,8-*p*-menthatriene	*Agathis atropurpurea*	[10]
1,5,9,9-tetramethyl-1,4,7-cycloundecatriene	*Agathis borneensis*	[11]
1,7,7-trimethyl-3-phenethylidenebicyclo[2.2.1]heptan-2-one	*Araucaria columnaris*	[75]
1,7-di-*ep*i-α-cedrenal	*Araucaria cunninghamii*	[81]
1*H*-*N*-hydrxynaphth(2,3)imidazole-6,7-dicarboximide	*Araucaria columnaris*	[74]
*1H*-*N*-hydroxynaphth(2,3-d)imidazole-6,7-dicarboximide	*Araucaria columnaris*	[74]
1,8-cineol	*Agathis robusta*, *Araucaria columnaris*, *Araucaria cunninghamii*, *Araucaria hunsteinii*, *Wollemia nobilis*	[8,81]
1-ethenyl-1-methyl-2,4-bis(1-methylethenyl) cyclohexane	*Wollemia nobilis*	[106]
1-heptacosanol	*Araucaria columnaris*	[75]
1-iodo-2-methylundecane	*Agathis borneensis*	[11]
1-octen-3-yl acetate	*Agathis atropurpurea*, *Araucaria cunninghamii*, *Araucaria heterophylla*	[10,81]
1-tridecene	*Araucaria cunninghamii*	[81]
2(1*H*)-phenanthrenone	*Agathis borneensis*	[11]
2,2-dimethyl-valeroyl chloride	*Wollemia nobilis*	[106]
2,3-di-amino-2-methylpropanoic acid	*Araucaria columnaris*	[74]
2,4-dimethyl-furan	*Araucaria columnaris*	[74]
2,3,3-trimethyl-octane	*Agathis borneensis*	[11]
2,4,6-trimethyl-octane	*Agathis borneensis*	[11]
2,5-dimethoxy-*p*-cymene	*Agathis atropurpurea*	[10]
2-acetyl-2-carene	*Wollemia nobilis*	[106]
2-pentyl-furan	*Agathis robusta*, *Araucaria cunninghamii*	[24,83]
3-4-methoxyphenyl-2-propenoic acid	*Araucaria columnaris*	[74]
3-ethyl-3-methyl-heptane	*Agathis borneensis*	[11]
3-methylene-1,7-octadiene	*Wollemia nobilis*	[106]
3-octanol	*Araucaria cunninghamii*	[81]
3-octanol acetate	*Araucaria cunninghamii*	[81]
**3-trimethylsilyloxypropyl hexadecanoate**	*Araucaria cunninghamii*	[84]
(3*Z*)-cembrene A	*Araucaria heterophylla*	[81]
(3*Z*)-hexenyl-2-methyl butanoate	*Araucaria cunninghamii*	[81]
(3*Z*)-hexenyl-butanoate	*Araucaria cunninghamii*	[81]
(3*Z*)-hexenyl hexanoate	*Araucaria cunninghamii*	[81]
4,4,5-trimethyl-2-hexene,3-ethyl-2,7-dimethyl octane	*Agathis borneensis*	[11]
4,11,11-trimethyl-8-methylene-bicyclo[7.2.0]undec-4-(*Z*)-ene	*Agathis borneensis*	[11]
4β,17-(acetoxy)-kauran-18-al	*Wollemia nobilis*	[106]
4-[[[[(1,2-dichloroethylidene) amino]oxy]carbonyl]amino]-methyl ester benzoic acid	*Araucaria columnaris*	[74]
4-methylene-2,8,8-trimethyl-2-vinyl-bicyclo[5.2.0]nonane	*Agathis borneensis*	[11]
5,15-rosadiene	*Agathis macrophylla*, *Agathis moorei*, *Araucaria luxurians*, *Araucaria montana*, *Araucaria muelleri*, *Araucaria scopulorum*	[8]
5-*neo*-cedranol	*Araucaria cunninghamii*	[81]
6-camphenol	*Wollemia nobilis*	[106]
6-dimethylcyclohexene	*Agathis borneensis*	[11]
6,6-dimethyl-2-methylene-bicyclo[2.2.1]-heptan-3-one	*Wollemia nobilis*	[106]
6-methoxy-2-methyl-2-phenyl-2H-1-benzopyran	*Araucaria columnaris*	[74]
6-methyl-5-hepten-2-one	*Agathis philippinensis*, *Araucaria cunninghamii*	[21,81]
7,11-dimethyl-3-methylene-1,6,10-dodecatriene	*Wollemia nobilis*	[106]
8a(2*H*)-phenanthrenol	*Agathis borneensis*	[11]
8-methylene	*Agathis borneensis*	[11]
8-oxo-*cis*-ocimene	*Wollemia nobilis*	[106]
9-*epi*-(*E*)-caryophyllene	*Agathis robusta*	[24]
9-*epi*-sclarene	*Araucaria heterophylla*	[63]
9-octadecenoic acid	*Araucaria columnaris*	[75]
9-octadecenoic acid methyl ester	*Araucaria cunninghamii*	[84]
10-*nor*-calamenen-10-one	*Agathis robusta*	[24]
13-*epi*-manool	*Araucaria heterophylla*	[81]
14-hydroxy-(*Z*)-caryophyllene	*Araucaria cunninghamii*	[81]
14-hydroxy-9-*epi*-(*E*)-caryophyllene	*Araucaria cunninghamii*	[81]
15-kaurene	*Araucaria angustifolia*, *Araucaria cunninghamii*, *Araucaria heterophylla*, *Araucaria muelleri*, *Wollemia nobilis*	[8,81]
15-phylocaldene	*Wollemia nobilis*	[106]
16-kaurene	*Agathis australis*, *Agathis atropurpurea*, *Agathis macrophylla*, *Agathis microstachya*, *Agathis moorei*, *Araucaria bidwilli*, *Araucaria columnaris*, *Araucaria cunninghamii*, *Araucaria heterophylla*, *Araucaria luxurians*, *Araucaria montana*, *Wollemia nobilis*	[8,81,106]
16-phyllocladanol	*Araucaria scopulorum*	[8]
16-phyllocladene	*Araucaria heterophylla*	[63]
(+)-4-carene	*Agathis dammara*	[12]
(+)-15-beyerene	*Araucaria araucana*	[63]
(−)-7,13-abietadine	*Araucaria bidwilli*	[63]
(−)-8(14),15-pimaradiene	*Araucaria heterophylla*	[63]
(−)-13-*epi*-manoyl-oxide	*Araucaria araucana*	[63]
(−)-16-atisirene	*Araucaria araucana*	[63]
(−)-16-kaurene	*Araucaria araucana*, *Araucaria bidwilli*, *Araucaria heterophylla*	[63]
(−)-α-cadinol	*Araucaria araucana*	[65]
(−)-α-copaene	*Araucaria araucana*, *Araucaria heterophylla*	[63]
(−)-β-caryophyllene	*Araucaria araucana*, *Araucaria heterophylla*	[63]
(−)-δ-cadinene	*Araucaria araucana*	[63]
(−)-atisirene	*Araucaria araucana*	[65,66]
(−)-germacrene D	*Araucaria heterophylla*	[63]
(−)-*iso*-atisirene	*Araucaria araucana*	[66]
(−)-*iso*-kaurene	*Araucaria araucana*	[66]
(−)-kaurene	*Araucaria araucana*	[65,66]
(−)-rosa-5,15-diene	*Araucaria araucana*	[63]
(−)-sandaracopimaradiene	*Araucaria heterophylla*	[63]
(−)-sclarene	*Araucaria araucana*	[63]
(−)-trachylobane	*Araucaria araucana*	[63,65,66]
[1*ar*-(1a.α,4.β,4a.β,7.α,7a.β,7b.α]-decahydro-1,1,4,7-tetramethyl-1H-cycloprop[e]azulen-4-ol	*Wollemia nobilis*	[106]
α-amorphene	*Araucaria cunninghamii*, *Araucaria heterophylla*	[81]
α-bisabolol	*Agathis dammara*	[12]
α-cadinene	*Araucaria heterophylla*	[81]
α-cadinol	*Agathis australis*, *Agathis microstachya*, *Agathis moorei*, *Araucaria angustifolia*, *Araucaria heterophylla*, *Wollemia nobilis*	[8,81,106]
α-calacorene	*Araucaria heterophylla*	[81]
α-campholenal	*Agathis robusta*, *Araucaria cunninghamii*	[24,83]
α-caryophyllene	*Agathis borneensis*	[11]
α-cedrene epoxide	*Agathis robusta*	[24]
α-copaene	*Agathis australis*, *Agathis atropurpurea*, *Agathis dammara*, *Agathis macrophylla*, *Agathis microstachya*, *Agathis moorei*, *Agathis ovata*, *Agathis philippinensis*, *Agathis robusta*, *Araucaria angustifolia*, *Araucaria bidwilli*, *Araucaria columnaris*, *Araucaria cunninghamii*, *Araucaria heterophylla*, *Araucaria hunsteinii*, *Araucaria luxurians*, *Araucaria montana*, *Araucaria muelleri*, *Araucaria scopulorum*, *Wollemia nobilis*	[8,12,21,24,63,81,83]
α-cubebene	*Agathis australis*, *Agathis atropurpurea*, *Agathis borneensis*, *Agathis macrophylla*, *Agathis microstachya*, *Agathis moorei*, *Agathis ovata*, *Agathis robusta*, *Araucaria bidwilli*, *Araucaria columnaris*, *Araucaria cunninghamii*, *Araucaria heterophylla*, *Araucaria hunsteinii*, *Araucaria luxurians*, *Araucaria montana*, *Araucaria scopulorum*	[8,11,24,81,83]
α-cubene	*Araucaria bidwilli*	[63]
α-fenchene	*Agathis atropurpurea*, *Agathis microstachya*, *Agathis philippinensis*, *Araucaria cunninghamii*	[8,21,81]
α-guaiene	*Araucaria heterophylla*	[81]
α-gurjunene	*Agathis australis*, *Agathis atropurpurea*, *Agathis microstachya*, *Agathis moorei*	[8]
α-humulene	*Agathis robusta*, *Araucaria cunninghamii*	[24,81]
α-muurolol	*Araucaria cunninghamii*	[81]
α-muurolene	*Agathis macrophylla*, *Agathis microstachya*, *Agathis moorei*, *Agathis robusta*, *Araucaria columnaris*, *Araucaria heterophylla*, *Araucaria hunsteinii*, *Araucaria montana*, *Araucaria scopulorum*, *Wollemia nobilis*	[8,24,81,106]
α-phellandrene	*Agathis dammara*, *Araucaria cunninghamii*,	[12,81]
α-pinene	*Agathis australis*, *Agathis atropurpurea*, *Agathis dammara*, *Agathis macrophylla*, *Agathis microstachya*, *Agathis moorei*, *Agathis philippinensis*, *Agathis robusta*, *Araucaria angustifolia*, *Araucaria bidwilli*, *Araucaria cunninghamii*, *Araucaria heterophylla*, *Araucaria hunsteinii*, *Araucaria luxurians*, *Araucaria montana*, *Araucaria scopulorum*, *Wollemia nobilis*	[8,12,21,24,81,83,87]
α-humulene	*Araucaria cunninghamii*, *Araucaria heterophylla*	[81,83]
α-muurolol	*Araucaria cunninghamii*, *Araucaria heterophylla*	[81]
α-phellandrene	*Araucaria cunninghamii*	[83]
α-selinene	*Agathis robusta*	[24]
α-terpinene	*Agathis atropurpurea*, *Agathis microstachya*, *Agathis robusta*, *Araucaria cunninghamii*, *Araucaria heterophylla*	[8,24,81,83]
α-terpineol	*Agathis atropurpurea*, *Agathis dammara*, *Agathis microstachya*, *Agathis philippinensis*, *Araucaria cunninghamii*, *Araucaria heterophylla*, *Araucaria montana*	[8,10,12,21,87]
α-terpinyl acetate	*Agathis robusta*	[24]
α-thujene	*Agathis philippinensis*, *Agathis robusta*, *Araucaria cunninghamii*	[8,21,24,81]
α-thujone	*Araucaria cunninghamii*	[83]
α-*trans*-bergamotene	*Araucaria heterophylla*	[81]
α-tricyclene	*Agathis dammara*	[12]
α-ylangene	*Araucaria heterophylla*	[81]
β-bisabolene	*Agathis atropurpurea*, *Agathis dammara*	[10,12]
β-bourbebene	*Araucaria hunsteinii*	[8]
β-bourbonene	*Agathis australis*, *Agathis macrophylla*, *Agathis moorei*, *Agathis ovata*, *Agathis robusta*, *Araucaria angustifolia*, *Araucaria columnaris*, *Araucaria heterophylla*, *Araucaria montana*, *Araucaria scopulorum*	[8,24,81]
β-calacorene	*Araucaria cunninghamii*	[83]
β-caryophyllene	*Agathis australis*, *Agathis atropurpurea*, *Agathis borneensis*, *Agathis macrophylla*, *Agathis microstachya*, *Agathis moorei*, *Agathis ovata*, *Agathis robusta*, *Araucaria angustifolia*, *Araucaria bidwilli*, *Araucaria columnaris*, *Araucaria cunninghamii*, *Araucaria heterophylla*, *Araucaria hunsteinii*, *Araucaria luxurians*, *Araucaria montana*, *Araucaria muelleri*, *Araucaria scopulorum*, *Wollemia nobilis*	[8,11,83,87]
β-chamigrene	*Agathis robusta*	[24]
β-copaen-4-α-ol	*Agathis robusta*	[24]
β-copaene	*Agathis australis*, *Agathis robusta*, *Araucaria angustifolia*, *Araucaria bidwilli*, *Araucaria cunninghamii*, *Araucaria heterophylla*	[8,24,81]
β-*cis*-ocimene	*Wollemia nobilis*	[106]
β-cubebene	*Agathis australis*, *Agathis atropurpurea,* *Agathis borneensis*, *Agathis macrophylla*, *Agathis microstachya*, *Agathis moorei*, *Araucaria bidwilli*, *Araucaria columnaris*	[8,11,63]
β-elemene	*Agathis macrophylla*, *Agathis microstachya*, *Araucaria angustifolia*, *Araucaria columnaris*, *Araucaria cunninghamii*, *Araucaria heterophylla*, *Araucaria hunsteinii*, *Araucaria muelleri*, *Araucaria scopulorum*	[8,81,83]
β-myrcene	*Agathis dammara*, *Agathis robusta*, *Wollemia nobilis*	[12,24,106]
β-ocimene	*Araucaria cunninghamii*	[81]
β-oplopenone	*Araucaria heterophylla*	[81]
β-panasinsene	*Araucaria cunninghamii*	[81]
β-phellandrene	*Agathis atropurpurea*, *Agathis microstachya*, *Araucaria hunsteinii*, *Wollemia nobilis*	[8]
β-pinene	*Agathis atropurpurea*, *Agathis dammara*, *Agathis microstachya*, *Agathis philippinensis*, *Agathis robusta*, *Araucaria angustifolia*, *Araucaria columnaris*, *Araucaria cunninghamii*, *Araucaria heterophylla*, *Araucaria hunsteinii*, *Araucaria luxurians*, *Araucaria montana*, *Araucaria scopulorum*, *Wollemia nobilis*	[8,12,21,24,81,83,87,106]
β-santalene	*Araucaria heterophylla*	[81]
β-selinene	*Agathis robusta*, *Araucaria cunninghamii*	[24,81,83]
β-terpineol	*Agathis philippinensis*	[21]
β-ylangene	*Agathis australis*, *Agathis atropurpurea*, *Agathis macrophylla*, *Agathis microstachya*, *Agathis moorei*, *Araucaria columnaris*, *Araucaria cunninghamii*, *Araucaria hunsteinii*, *Araucaria muelleri*, *Araucaria scopulorum*	[8]
γ-cadinene	*Agathis robusta*, *Araucaria araucana*, *Araucaria cunninghamii*, *Araucaria heterophylla*	[24,65,81,83]
γ-elemene	*Agathis microstachya*, *Araucaria scopulorum*	[8]
γ-gurjunene	*Araucaria heterophylla*	[81]
γ-muurolene	*Agathis robusta*, *Araucaria cunninghamii*	[24,83]
γ-terpinene	*Agathis atropurpurea*, *Agathis dammara*, *Agathis microstachya*, *Agathis philippinensis*, *Araucaria cunninghamii*, *Araucaria heterophylla*, *Wollemia nobilis*	[8,10,12,21,81,83]
δ-3-carene	*Araucaria cunninghamii*	[81,83]
δ-amorphene	*Araucaria heterophylla*	[81]
δ-cadinene	*Agathis australis*, *Agathis atropurpurea*, *Agathis borneensis*, *Agathis dammara*, *Agathis macrophylla*, *Agathis microstachya*, *Agathis moorei*, *Agathis ovata*, *Agathis robusta*, *Araucaria angustifolia*, *Araucaria bidwilli*, *Araucaria columnaris*, *Araucaria cunninghamii*, *Araucaria heterophylla*, *Araucaria luxurians*, *Araucaria montana*, *Araucaria muelleri*, *Araucaria scopulorum*, *Wollemia nobilis*	[8,11,12,24,63,81,83]
δ-cadinol	*Agathis microstachya*, *Agathis moorei*	[8]
δ-carene	*Wollemia nobilis*	[106]
δ-elemene	*Agathis robusta*, *Araucaria scopulorum*	[8,24]
δ-terpineol	*Araucaria cunninghamii*	[83]
abietadiene	*Araucaria heterophylla*	[81]
abietate	*Agathis borneensis*	[11]
abietatriene	*Araucaria cunninghamii*, *Araucaria heterophylla*	[81]
acetophenone	*Araucaria cunninghamii*	[81]
alkyl chains	*Agathis australis*, *Agathis atropurpurea*, *Agathis macrophylla*, *Agathis microstachya*, *Agathis moorei*, *Agathis ovata*, *Agathis robusta*, *Araucaria angustifolia*, *Araucaria bidwilli*, *Araucaria columnaris*, *Araucaria cunninghamii*, *Araucaria heterophylla*, *Araucaria luxurians*, *Araucaria montana*, *Araucaria muelleri*, *Araucaria scopulorum*, *Wollemia nobilis*	[8]
*allo*-aromadendrene	*Agathis australis*, *Agathis microstachya*, *Agathis moorei*, *Agathis robusta*, *Araucaria angustifolia*, *Araucaria bidwilli*, *Araucaria columnaris*, *Araucaria cunninghamii*, *Araucaria heterophylla*, *Araucaria luxurians*, *Araucaria montana*, *Araucaria muelleri*, *Araucaria scopulorum*, *Wollemia nobilis*	[8,24,81,83]
androstenone	*Agathis borneensis*	[11]
*ar*-turmerone	*Araucaria cunninghamii*	[83]
arachidonic acid	*Araucaria cunninghamii*	[84]
aristolochene	*Araucaria heterophylla*	[81]
aromadendrene	*Agathis australis*, *Agathis macrophylla*, *Agathis microstachya*, *Agathis moorei*, *Agathis robusta*, *Araucaria angustifolia*, *Araucaria bidwilli*, *Araucaria columnaris*, *Araucaria cunninghamii*, *Araucaria heterophylla*, *Araucaria hunsteinii*, *Araucaria luxurians*, *Araucaria montana*, *Araucaria muelleri*, *Araucaria scopulorum*, *Wollemia nobilis*	[8,24,81,83]
aromadendrene (2)-oxide	*Wollemia nobilis*	[106]
benzoic acid	*Araucaria columnaris*	[74]
bergamol	*Wollemia nobilis*	[106]
beyerene	*Araucaria cunninghamii*	[83]
bicycloelemene	*Agathis australis*, *Agathis atropurpurea*, *Agathis macrophylla*, *Agathis microstachya*, *Araucaria angustifolia*, *Araucaria columnaris*, *Araucaria luxurians*, *Araucaria muelleri*	[8]
bicyclogermacrene	*Agathis australis*, *Agathis atropurpurea*, *Agathis microstachya*, *Araucaria angustifolia*, *Araucaria bidwilli*, *Araucaria columnaris*, *Araucaria cunninghamii*, *Araucaria heterophylla*, *Araucaria hunsteinii*, *Araucaria luxurians*, *Araucaria montana*, *Araucaria muelleri*, *Araucaria scopulorum*, *Wollemia nobilis*	[8,81,83]
borneol	*Agathis robusta*	[24]
bornyl acetate	*Agathis robusta*	[24]
bicetyl	*Agathis borneensis*	[11]
bornyl acetate	*Agathis atropurpurea*, *Wollemia nobilis*	[10,106]
calacorene	*Agathis australis*, *Agathis macrophylla*, *Agathis moorei*, *Araucaria columnaris*, *Araucaria cunninghamii*, *Araucaria hunsteinii*, *Araucaria scopulorum*	[8]
calamenene	*Agathis macrophylla*, *Agathis microstachya*, *Agathis moorei*, *Agathis ovata*, *Araucaria columnaris*, *Araucaria hunsteinii*, *Araucaria luxurians*, *Araucaria scopulorum*	[8]
camphene	*Agathis atropurpurea*, *Agathis australis*, *Agathis dammara*, *Agathis microstachya*, *Agathis philippinensis*, *Agathis robusta*, *Araucaria cunninghamii*, *Araucaria heterophylla*, *Araucaria hunsteinii*, *Wollemia nobilis*	[8,10,12,21,24,81,83,87]
campherenone	*Araucaria cunninghamii*	[83]
carotol	*Agathis robusta*	[24]
carvone	*Agathis atropurpurea*, *Agathis philippinensis*, *Agathis robusta*, *Wollemia nobilis*	[10,21,24,106]
cariophyllene	*Araucaria columnaris*	[75]
caryophyllene oxide	*Agathis borneensis*, *Agathis macrophylla*, *Agathis microstachya*, *Agathis moorei*, *Agathis ovata*, *Agathis robusta*, *Araucaria bidwilli*, *Araucaria columnaris*, *Araucaria cunninghamii*, *Araucaria heterophylla*, *Araucaria luxurians*, *Araucaria montana*, *Araucaria muelleri*, *Araucaria scopulorum*, *Wollemia nobilis*	[8,11,24,81,83,106]
cedr-8-en-15-ol	*Araucaria columnaris*	[75]
cetane	*Agathis borneensis*	[11]
cholesterol	*Araucaria cunninghamii*	[84]
*cis*-β-terpineol	*Agathis atropurpurea*	[10]
*cis*-*p*-mentha-1,8-dien-6-ol	*Agathis philippinensis*	[21]
*cis*-*p*-mentha-1(7),8-dien-2-ol	*Agathis philippinensis*	[21]
*cis*-pinocarvyl acetate	*Agathis robusta*	[24]
*cis*-sabinene hydrate	*Agathis philippinensis*	[21]
*cis*-thujopsenal	*Araucaria cunninghamii*	[81]
*cis*-thujopsene	*Agathis robusta*	[24]
copaene	*Agathis borneensis*	[11]
cubenol	*Agathis australis*, *Agathis microstachya*, *Araucaria bidwilli*, *Araucaria columnaris*, *Araucaria hunsteinii*, *Araucaria luxurians*, *Araucaria scopulorum*	[8]
cubitene	*Araucaria heterophylla*	[81]
cyclobuta[1,2:3,4]dicyclopentene decahydro-3a-methyl-6-methylene-1-(1-methylethyl)-[1S-(1.α,3a.α,3b.β,6a.β,6b.α)]	*Wollemia nobilis*	[106]
cyclocolorenone	*Araucaria cunninghamii*	[81]
cyclohexene	*Agathis borneensis*	[11]
**cyclononasiloxane**	*Araucaria cunninghamii*	[84]
dehydro-1,8-cineol	*Agathis atropurpurea*	[10]
dihydro-α-terpineol	*Agathis philippinensis*	[21]
dehydro-abeitol	*Araucaria cunninghamii*	[81]
dihydro-carveol	*Agathis atropurpurea*	[10]
dihydro-carvyl acetate	*Agathis robusta*	[24]
dodecane	*Agathis borneensis*	[11]
di-isooctyl adipate	*Araucaria cunninghamii*	[84]
docosahexaenoic acid	*Araucaria cunninghamii*	[84]
docosyl acetate	*Araucaria columnaris*	[75]
dolabradiene	*Araucaria cunninghamii*, *Araucaria heterophylla*	[63,81]
(*E*)-β-farnesene	*Araucaria cunninghamii*, *Araucaria heterophylla*	[81]
(*E*)-β-ocimene	*Agathis philippinensis*, *Araucaria cunninghamii*	[21,81]
(*E*)-3(10)-caren-2-ol	*Wollemia nobilis*	[106]
(*E*)-3(10)-caren-4-ol	*Wollemia nobilis*	[106]
(*E*)-α-ionone	*Agathis robusta*	[24]
*E*-biformene	*Araucaria bidwilli*	[63]
(*E*)-caryophyllene	*Agathis robusta*, *Araucaria cunninghamii*, *Araucaria heterophylla*	[24,81]
(*E*)-myroxide	*Agathis robusta*	[24]
(*E*)-nerolidol	*Araucaria heterophylla*	[81]
(*E*)-9-octadecenoic acid ethyl ester	*Araucaria cunninghamii*	[84]
**eicosamethyl-cyclodecasiloxane**	*Araucaria cunninghamii*	[84]
eicosane	*Agathis borneensis*	[11]
*ent*-rosa-5,15-diene	*Araucaria heterophylla*	[81]
*ep*i-α-cadinol	*Araucaria heterophylla*	[81]
*ep*i-α-cedrenal	*Araucaria heterophylla*	[81]
*epi*-α-selinene	*Agathis robusta*	[24]
*epi*-cubenol	*Agathis australis*, *Agathis atropurpurea*, *Agathis macrophylla*, *Agathis microstachya*, *Araucaria columnaris*, *Araucaria hunsteinii*, *Araucaria luxurians*, *Araucaria scopulorum*	[8]
*epi*-cyclocolorenone	*Araucaria cunninghamii*	[81]
*epi*-globulol	*Araucaria scopulorum*	[8]
*epi*-laurenene	*Araucaria heterophylla*	[81]
*epi*-zonarene	*Araucaria heterophylla*	[63]
ethyl 9-octadecenoate	*Araucaria cunninghamii*	[84]
ethyl heptadecanoate	*Araucaria cunninghamii*	[84]
farnesane	*Agathis borneensis*	[11]
farnesol	*Agathis borneensis*	[11]
fenchone	*Agathis philippinensis*	[21]
filipendulal	*Wollemia nobilis*	[106]
geraniolene	*Araucaria araucana*	[65,66]
germacrene B	*Agathis robusta*, *Araucaria heterophylla*, *Wollemia nobilis*	[24,81,106]
germacrene D	*Agathis australis*, *Agathis atropurpurea*, *Agathis borneensis, Agathis dammara*, *Agathis macrophylla*, *Agathis microstachya*, *Agathis moorei*, *Agathis ovata*, *Agathis robusta*, *Araucaria angustifolia*, *Araucaria bidwilli*, *Araucaria columnaris*, *Araucaria cunninghamii*, *Araucaria heterophylla*, *Araucaria hunsteinii*, *Araucaria luxurians*, *Araucaria muelleri*, *Araucaria scopulorum*, *Wollemia nobilis*	[8,11,12,63,81,83,106]
globulol	*Agathis australis*, *Agathis atropurpurea*, *Agathis microstachya*, *Agathis moorei*, *Agathis robusta*, *Araucaria angustifolia*, *Araucaria bidwilli*, *Araucaria columnaris*, *Araucaria cunninghamii*, *Araucaria heterophylla*, *Araucaria hunsteinii*, *Araucaria luxurians*, *Araucaria montana*, *Araucaria muelleri*, *Araucaria scopulorum*, *Wollemia nobilis*	[8,81]
heptacosane	*Agathis borneensis*	[11]
hexyl butanoate	*Araucaria cunninghamii*	[81]
hexyl *iso*-valerate	*Araucaria cunninghamii*	[81]
hibaene	*Agathis australis*, *Agathis ovata*, *Araucaria angustifolia*, *Araucaria araucana*, *Araucaria bidwilli*, *Araucaria columnaris*, *Araucaria cunninghamii*, *Araucaria heterophylla*, *Wollemia nobilis*	[8,65,66,81]
humulene	*Agathis australis*, *Agathis macrophylla*, *Agathis microstachya*, *Agathis moorei*, *Agathis ovata*, *Agathis robusta*, *Araucaria angustifolia*, *Araucaria bidwilli*, *Araucaria columnaris*, *Araucaria cunninghamii*, *Araucaria heterophylla*, *Araucaria hunsteinii*, *Araucaria luxurians*, *Araucaria montana*, *Araucaria muelleri*, *Araucaria scopulorum,* *Wollemia nobilis*	[8]
humulene epoxide II	*Agathis robusta*, *Araucaria cunninghamii*	[24,81,83]
icosane	*Agathis borneensis*	[11]
intermedeol	*Agathis robusta*	[24]
*iso*-aromadendrene epoxide	*Wollemia nobilis*	[106]
*iso*-bornyl acetate	*Agathis robusta*	[24]
*iso*-geraniol	*Wollemia nobilis*	[106]
*iso*-longifolene	*Araucaria heterophylla*	[81]
*iso*-pimara-9(11),15-diene	*Araucaria heterophylla*	[81]
kaur-16-en-18-oic acid methyl ester	*Wollemia nobilis*	[106]
kaur-16-en-19-ol	*Araucaria columnaris*	[75]
kaurene	*Araucaria cunninghamii*	[83]
*L*-valine-*N*-[*N*-[*N*2,*N*6-*b*i*s*-(1-oxodecyl)-*L*-lysyl]glycyl]-methyl ester	*Araucaria cunninghamii*	[84]
laurenan-2-one	*Araucaria cunninghamii*	[81]
laurenene	*Araucaria cunninghamii*, *Araucaria heterophylla*	[81,83]
ledol	*Araucaria hunsteinii*, *Araucaria scopulorum*	[8]
limonen-10-ol	*Agathis philippinensis*	[21]
limonene	*Agathis australis*, *Agathis atropurpurea*, *Agathis dammara*, *Agathis macrophylla*, *Agathis microstachya*, *Agathis moorei*, *Agathis ovata*, *Agathis philippinensis*, *Agathis robusta*, *Araucaria angustifolia*, *Araucaria araucana*, *Araucaria bidwilli*, *Araucaria cunninghamii*, *Araucaria heterophylla*, *Araucaria hunsteinii*, *Araucaria luxurians*, *Araucaria montana*, *Araucaria muelleri*, *Wollemia nobilis*	[8,10,11,12,21,65,81,87]
longipinanol	*Araucaria cunninghamii*	[81]
luxuriadiene	*Agathis australis*, *Agathis atropurpurea*, *Agathis macrophylla*, *Araucaria columnaris*, *Araucaria cunninghamii*, *Araucaria heterophylla*, *Araucaria luxurians*, *Araucaria montana*, *Araucaria muelleri*, *Araucaria scopulorum*	[8,81]
*m*-cymenene	*Agathis atropurpurea*	[10]
manool	*Araucaria columnaris*	[75]
manool oxide	*Araucaria heterophylla*	[81]
menthol	*Araucaria cunninghamii*	[83]
**methyl-β-D-mannofuranoside**	*Agathis borneensis*	[11]
methyl-chavicol	*Agathis robusta*	[24]
methyl-eugenol	*Araucaria hunsteinii*	[8]
methyl-isobutyrate	*Agathis borneensis*	[11]
methyl-(*Z*)-5,11,14,17-eicosatetraenoate	*Araucaria columnaris*	[75]
myrcene	*Agathis atropurpurea*, *Agathis macrophylla*, *Agathis microstachya*, *Agathis moorei*, *Araucaria angustifolia*, *Araucaria bidwilli*, *Araucaria cunninghamii*, *Araucaria heterophylla*, *Araucaria hunsteinii*, *Araucaria montana*, *Araucaria muelleri*, *Araucaria scopulorum*, *Wollemia nobilis*	[8]
myrtenol	*Agathis robusta*, *Araucaria cunninghamii*, *Wollemia nobilis*	[24,81,83,106]
myrtenyl acetate	*Agathis robusta*	[24]
*N*,*N*-*bis*(2-hydroxyethyl)dodecanamide	*Araucaria columnaris*	[75]
*n*-docosane	*Agathis borneensis*	[11]
*n*-heptadecane	*Agathis borneensis*	[11]
*n*-hexacosane	*Agathis borneensis*	[11]
*n*-nonadecane	*Agathis borneensis*	[11]
*n*-nonane	*Araucaria cunninghamii*, *Araucaria heterophylla*	[11]
*n*-octacosane	*Agathis borneensis*	[11]
*n*-pentacosane	*Agathis borneensis*	[11]
*n*-pentadecane	*Agathis borneensis*	[11]
*n*-pentadecanoic acid	*Agathis borneensis*	[11]
*n*-tetradecane	*Agathis borneensis*	[11]
*n*-tetratriacontane	*Agathis borneensis*	[11]
*n*-triacontane	*Agathis borneensis*	[11]
*n*-tridecane	*Araucaria cunninghamii*	[11]
*n*-undecane	*Araucaria cunninghamii*, *Araucaria heterophylla*	[11]
naphthalene	*Agathis borneensis*	[11]
neral	*Agathis philippinensis*	[21]
nonane	*Araucaria cunninghamii*	[83]
*nor*-pristane	*Agathis borneensis*	[11]
*o*-cymene	*Agathis dammara*	[12]
*O*-ethyl-hydroxylamine	*Araucaria cunninghamii*	[84]
occidentalol	*Agathis robusta*, *Araucaria cunninghamii*	[24,81]
occidentalol acetate	*Agathis robusta*, *Araucaria cunninghamii*	[24,81]
octadecane	*Agathis borneensis*	[11]
**octadecyl iodide**	*Agathis borneensis*	[11]
octane	*Agathis borneensis*	[11]
octen-1-ol acetate	*Wollemia nobilis*	[106]
octyl ether	*Agathis borneensis*	[11]
*p*-cymen-8-ol	*Agathis macrophylla*, *Agathis robusta*, *Agathis philippinensis*, *Araucaria cunninghamii*	[8,21,24]
*p*-cymene	*Agathis macrophylla*, *Agathis microstachya*, *Agathis moorei*, *Agathis philippinensis*, *Agathis robusta*, *Araucaria angustifolia*, *Araucaria bidwilli*, *Araucaria cunninghamii*, *Araucaria heterophylla*, *Araucaria hunsteinii*, *Araucaria luxurians*, *Araucaria montana*, *Araucaria muelleri*, *Wollemia nobilis*	[8,21,24,81]
*p*-cymenene	*Agathis robusta*	[24]
*p*-menth-8-en-2-ol acetate	*Wollemia nobilis*	[106]
*p*-mentha-1,4-dien-7-ol	*Araucaria cunninghamii*	[83]
*p*-mentha-3,8-diene	*Agathis robusta*	[24]
*p*-mentha-6,8-dien-2-ol acetate	*Wollemia nobilis*	[106]
palmitic acid	*Agathis borneensis*, *Araucaria columnaris*	[11,75]
palmitic acid ethyl ester	*Araucaria* *cunninghamii*	[84]
palustrol	*Agathis australis*, *Agathis macrophylla*, *Agathis microstachya*, *Araucaria angustifolia*, *Araucaria columnaris*, *Araucaria cunninghamii*, *Araucaria luxurians*	[8]
phyllocladanol	*Araucaria heterophylla*	[81]
phyllocladene	*Agathis atropurpurea*, *Agathis microstachya*, *Agathis ovata*, *Araucaria angustifolia*, *Araucaria cunninghamii*, *Araucaria heterophylla*, *Araucaria montana*, *Araucaria muelleri*	[8,81,83]
pimaradiene	*Araucaria cunninghamii*, *Araucaria heterophylla*	[81]
pinocarvone	*Agathis robusta*	[24]
rimuene	*Agathis robusta*, *Araucaria heterophylla*	[8,24,81]
sabinene	*Agathis atropurpurea*, *Agathis dammara*, *Agathis microstachya*, *Agathis philippinensis*, *Agathis robusta*, *Araucaria cunninghamii*, *Araucaria heterophylla*, *Araucaria hunsteinii*, *Wollemia nobilis*	[8,12,21,24,81,83]
sandaracopimara-8(14),15-diene	*Araucaria heterophylla*	[81]
sandaracopimar-15-en-8.b.-yl acetate	*Wollemia nobilis*	[106]
sandaracopimarinol	*Araucaria cunninghamii*, *Araucaria heterophylla*	[81]
sativene	*Araucaria cunninghamii*, *Wollemia nobilis*	[81,106]
sclarene	*Agathis australis*, *Agathis ovata*, *Araucaria bidwilli*, *Araucaria columnaris*, *Araucaria heterophylla*, *Araucaria hunsteinii*, *Araucaria muelleri*, *Araucaria scopulorum*	[8,63,81]
sesquisabinene B	*Araucaria cunninghamii*	[83]
shyobunol	*Araucaria cunninghamii*	[83]
sorbaldehyde	*Agathis borneensis*	[11]
squamulosone	*Araucaria cunninghamii*	[81]
spathulenol	*Agathis australis*, *Agathis atropurpurea*, *Agathis macrophylla*, *Agathis microstachya*, *Agathis moorei*, *Agathis ovata*, *Agathis robusta*, *Araucaria angustifolia*, *Araucaria bidwilli*, *Araucaria columnaris*, *Araucaria cunninghamii*, *Araucaria heterophylla*, *Araucaria hunsteinii*, *Araucaria luxurians*, *Araucaria montana*, *Araucaria muelleri*, *Araucaria scopulorum*, *Wollemia nobilis*	[8,24,81,83,106]
T-cadinol	*Agathis australis, Agathis microstachya*, *Agathis moorei*, *Araucaria columnaris*, *Araucaria hunsteinii*, *Araucaria scopulorum*	[8]
T-muurolol	*Agathis australis*, *Agathis microstachya*, *Agathis moorei*, *Araucaria cunninghamii*, *Araucaria hunsteinii*	[8,83]
T(*Z*)-β-ocimene	*Araucaria cunninghamii*	[83]
terpinen-4-ol	*Agathis dammara*, *Agathis philippinensis*, *Agathis robusta*, *Araucaria cunninghamii*	[12,21,24,81,83]
terpinene	*Wollemia nobilis*	[8]
terpinolene	*Agathis atropurpurea*, *Agathis dammara*, *Agathis microstachya*, *Agathis philippinensis*, *Araucaria cunninghamii*, *Araucaria heterophylla*, *Araucaria hunsteinii*, *Wollemia nobilis*	[8,10,12,21,81]
*tert*-butoxy 2 ethoxyethane	*Araucaria columnaris*	[74]
tetrahydro-geranyl acetate	*Wollemia nobilis*	[106]
thiophene	*Agathis borneensis*	[11]
thuja-2,4(10)-diene	*Agathis robusta*	[24]
*trans*-β-terpineol	*Agathis robusta*	[24]
*trans*-calamenene	*Agathis robusta*	[24]
*trans*-carveol	*Agathis robusta*, *Araucaria cunninghamii*	[24,83]
*trans*-phytol	*Agathis borneensis*	[11]
*trans*-longipinocarveol	*Wollemia nobilis*	[106]
*trans*-muurola-4(14),5-diene	*Araucaria heterophylla*	[81]
*trans*-*p*-mentha-1(7),8-dien-2-ol	*Agathis philippinensis*	[21]
*trans*-*p*-mentha-1,8-dien-6-ol	*Agathis philippinensis*	[21]
*trans*-pinocarveol	*Agathis philippinensis*	[21]
*trans*-pinocarvyl acetate	*Agathis robusta*	[24]
*trans*-piperitol	*Agathis philippinensis*	[21]
*trans*-sabinene hydrate	*Agathis robusta*	[24]
*trans*-sabinene hydrate acetate	*Agathis robusta*	[24]
*trans*-sabinol	*Agathis robusta*	[24]
*tran*s-*Z*-α-bisabolene epoxide	*Wollemia nobilis*	[106]
tricosyl acetate	*Araucaria columnaris*	[75]
tricyclene	*Agathis australis*, *Agathis atropurpurea*, *Agathis philippinensis*, *Araucaria cunninghamii*	[8,10,21,81,83]
undecane	*Araucaria cunninghamii*	[83]
untriacontane	*Agathis borneensis*	[11]
verbenol	*Wollemia nobilis*	[106]
verbenone	*Agathis robusta*, *Araucaria cunninghamii*, *Wollemia nobilis*	[24,83,106]
viridiflorene	*Agathis australis*, *Agathis microstachya*, *Agathis moorei*, *Agathis ovata*, *Agathis robusta*, *Araucaria angustifolia*, *Araucaria bidwilli*, *Araucaria columnaris*, *Araucaria heterophylla*, *Araucaria hunsteinii*, *Araucaria luxurians*, *Araucaria montana*, *Araucaria scopulorum*	[8,81]
viridiflorol	*Agathis australis*, *Agathis atropurpurea*, *Agathis microstachya*, *Agathis moorei*, *Agathis robusta*, *Araucaria angustifolia*, *Araucaria bidwilli*, *Araucaria columnaris*, *Araucaria cunninghamii*, *Araucaria heterophylla*, *Araucaria hunsteinii*, *Araucaria luxurians*, *Araucaria montana*, *Araucaria muelleri*, *Araucaria scopulorum*, *Wollemia nobilis*	[8,81]
(*Z*)-2-decenal	*Wollemia nobilis*	[106]
(*Z*)-β-farnesene	*Agathis robusta*	[24]
(*Z*)-β-ocimene	*Araucaria cunninghamii*	[81]
*Z*-β-terpineol	*Wollemia nobilis*	[106]
*Z*-biformene	*Araucaria bidwilli*	[63]

**Table 5:** Occurrence of polar fraction metabolites in Araucariaceae species.

Compound	Occurrence in the Genera	References
1,2-di-palmitoleoyl-3-myristoyl-*sn*-glycerol	*Wollemia* *nobilis*	[29]
1,3,4,5-tetrahydroxy-cyclohexane-carboxylic acid	*Araucaria angustifolia*	[48]
1,5,8,11,14,17-eicosapentaenoicacid	*Agathis robusta*	[25]
2α,3α,19α-trihydroxyurs-12-en-28-oic acid	*Agathis macrophylla*	[17]
2α,3β,19α-trihydroxyurs-12-en-28-oic acid	*Agathis macrophylla*	[17]
2α,3β- dihydroxyurs-12-en-28-oic acid	*Agathis macrophylla*	[17]
2α-hydroxy-8(14),15-sandaracopimaradien-18-oic acid	*Wollemia nobilis*	[31]
(2*S*)-1,2-di-*O*-[(9*Z*,12*Z*,15*Z*)-octadeca-9,12,15-trienoyl]-3-*O*-β-D- galactopyranosyl glycerol	*Agathis robusta*	[22]
2-*O*-acetyl-11-keto-boswellic acid	*Araucaria bidwilli*	[69]
3α-hydroxy-(13*S*)-l6-*nor*-pimar-7-en-l5-oic acid	*Agathis macrophylla*	[18]
3β,22,23- trihydroxystigmast-5-en-7-one	*Agathis macrophylla*	[17]
3β-hydroxymegastigman-5-en-9-*O*-β-D-glucopyranoside	*Agathis macrophylla*	[17]
3β-hydroxystigmast-6-one	*Agathis macrophylla*	[17]
3-*O*-methyl-D-chiroinositol	*Araucaria angustifolia*	[48]
3-glucoside-dihydro-quercetin	*Araucaria angustifolia*	[48]
4(15)-eudesmene-1β,6α-diol	*Agathis macrophylla*	[17]
4′-*O*-methyl-scutellarein	*Wollemia nobilis*	[30]
(4*S*,5*R*,9*S*,10*R*)-methyl-19-hydroxy-15,16-dinorlabda-8(17),11-*E*-dien-13-oxo-18-oate	*Agathis macrophylla*	[16]
(4*R*,5*R*,9*R*,10*R*,13*R*)-13-hydroxypodocarp-8(14)-en-19-oic acid	*Agathis macrophylla*	[16]
(4*R*,5*R*,9*R*,10*R*,13*S*)-13-hydroxypodocarp-8(14)-en-19-oic acid	*Agathis macrophylla*	[16]
4-hydroxy-benzaldehyde	*Araucaria angustifolia*	[58]
4-nitrophenyl-β-D-glucopyranoside	*Araucaria angustifolia*	[48]
4′-methoxy-tectorigenin	*Araucaria angustifolia*	[48]
4-*n*-butoxylphenylpropanetriol	*Araucaria columnaris*	[80]
5-(*E*)-coumaroyloxy-quinic acid *n*-butyl ester	*Araucaria cunninghamii*	[79]
5-(*Z*)-coumaroyloxyquinic acid *n*-butyl ester	*Araucaria cunninghamii*	[79]
5-methoxy-lariciresinol-9-acetate	*Araucaria angustifolia*	[58]
5′-methoxy-lariciresinol-9-acetate	*Araucaria angustifolia*	[58]
5-methoxy-pinoresinol	*Araucaria angustifolia*	[58]
5-methoxy-eudesmin	*Araucaria angustifolia*	[58]
5-*p*-*cis*-coumaroyl-quinic acid	*Araucaria columnaris*	[80]
5-*p*-*trans*-coumaroyl-quinic acid	*Araucaria columnaris*	[80]
6′-*O*-acetyl-pina-2-ene-4,10-diol-10-*O*-β-D-glucopyranoside	*Wollemia nobilis*	[29]
(6*R*,9*S*)-3-oxo-α-ionol-9-*O*-β-D-glucopyranoside	*Araucaria columnaris*	[80]
(6*S*,9*R*)-roseoside	*Agathis macrophylla*	[17]
(6*S*,9*S*)-roseoside	*Araucaria columnaris*	[80]
7α,15α-dihydroxystigmast-4-en-3-one	*Agathis macrophylla*	[17]
7-hydroxy-labda-8(17),13(16),14-trien-19-yl-7′-*O*-methyl-(*E*)- coumarate	*Araucaria bidwilli*	[69]
7-hydroxy-labda-8(17),13(16),14-trien-19-yl-7′-*O*-methyl-(*Z*)- coumarate	*Araucaria bidwilli*	[69]
7-hydroxy-labda-8(17),13(16),14-trien-19-yl-(*E*)-coumarate	*Araucaria bidwilli*	[69]
7-hydroxy-labda-8(17),13(16),14-trien-19-yl-(*Z*)-coumarate	*Araucaria bidwilli*	[69]
7′-hydroxy-lariciresinol	*Araucaria angustifolia*	[58]
7′-hydroxy-lariciresinol-9-acetate	*Araucaria angustifolia*	[58]
7′-methoxy-lariciresinol	*Araucaria angustifolia*	[58]
7′-methoxy-lariciresinol-9-acetate	*Araucaria angustifolia*	[58]
7-oxocallitrisic acid	*Araucaria bidwilli*	[69]
7-*O*-methyl-agathisflavone	*Agathis alba*, *Araucaria araucana*, *Araucaria bidwilli*, *Araucaria columnaris*, *Araucaria cunninghamii*, *Araucaria rulei*, *Wollemia nobilis*	[6,7,29,64,70,89,107]
7”-*O*-methyl-amentoflavone	*Araucaria araucana*, *Araucaria columnaris*, *Araucaria cunninghamii*,	[64,70]
7-*O*-methyl-cupressuflavone	*Agathis alba*, *Agathis atropurpurea*, *Agathis australis*, *Agathis ovata*, *Agathis robusta*, *Araucaria bidwilli*, *Wollemia nobilis*	[6,7,9,31,70]
7′’-*O*-methyl-agathisflavone	*Agathis alba*, *Agathis atropurpurea*, *Agathis australis*, *Agathis ovata*, *Agathis robusta*, *Wollemia nobilis*	[6,9,22,31,33]
7′’-*O*-methyl-robustaflavone	*Araucaria angustifolia*	[56]
4′,7′’-di-*O*-methyl-agathisflavone	*Araucaria bidwilli*	[68]
4′,4′’-di-*O*-methyl-amentoflavone	*Araucaria bidwilli*	[68]
4′,4′’’-di-*O*-methyl-amentoflavone	*Araucaria angustifolia*	[57]
4′,4′’’-di-*O*-methyl-cupressuflavone	*Agathis macrophylla*	[17]
7,4′-di-*O*-methyl-amentoflavone	*Araucaria cunninghamii*	[70]
7,4′’’-di-*O*-methyl-agathisflavone	*Agathis alba*, *Araucaria columnaris*, *Araucaria cunninghamii*, *Araucaria rulei*, *Wollemia nobilis*	[7,28,29,33,70,89,107]
7,7′’-di-*O*-methyl-agathisflavone	*Agathis alba*, *Agathis atropurpurea*, *Agathis australis*, *Agathis robusta*, *Agathis ovata*, *Araucaria bidwilli*, *Araucaria columnaris*	[6,9,70]
7,7′’-di-*O*-methyl-amentoflavone	*Araucaria columnaris*	[70]
7,7′’-di-*O*-methyl-cupressuflavone	*Agathis alba*, *Agathis atropurpurea*, *Agathis australis*, *Agathis ovata*, *Agathis robusta*, *Araucaria araucana*, *Araucaria bidwilli*, *Araucaria cunninghamii*, *Araucaria rulei*, *Wollemia nobilis*	[6,7,9,64,70,89,107]
7,4′,7′’-tri-*O*-methyl-agathisflavone	*Agathis atropurpurea*, *Agathis australis*, *Agathis ovata*, *Araucaria bidwilli*	[9,69]
7,4′,4′’’-tri-*O*-methyl-amentoflavone	*Araucaria angustifolia*	[57]
7,4′,4′’’-tri-*O*-methyl-agathisflavone	*Wollemia nobilis*	[29,32,107]
7,4′,7′’-tri-*O*-methyl-cupressuflavone	*Agathis atropurpurea*, *Agathis australis*, *Agathis ovata*, *Araucaria columnaris*, *Araucaria cunninghamii*, *Wollemia nobilis*	[9,69,70,80,107]
7,7′’,4′’’-tri-*O*-methyl-agathisflavone	*Araucaria columnaris*	[70]
7,7′’,4′’’’-tri-*O*-methyl-cupressuflavone	*Araucaria rulei*	[89]
7, 4′,7′’-tri-*O*-methyl-amentoflavone	*Araucaria angustifolia*, *Araucaria columnaris*	[57,70]
7,7′’,4′’’-tri-*O*-methyl-amentoflavone	*Wollemia nobilis*	[29]
7,4′,7′’,4′’’-tetra-*O*-methyl-agathisflavone	*Agathis australis*, *Agathis macrophylla*, *Agathis ovata*, *Wollemia nobilis*	[9,17,107]
7,4′,7′’,4′’’-tetra-*O*-methyl-amentoflavone	*Araucaria angustifolia*, *Araucaria columnaris*, *Araucaria cunninghamii*, *Araucaria rulei*, *Wollemia nobilis*	[48,70,89,107]
7,4′,7′’,4′’’-tetra-*O*-methyl-cupressuflavone	*Agathis australis*, *Agathis ovata*, *Araucaria columnaris*, *Araucaria cunninghamii*, *Araucaria rulei*, *Wollemia nobilis*	[9,70,89,107]
7,4′,7′’,4′’’-tetra-*O*-methyl-robustaflavone	*Wollemia nobilis*	[29]
7-*O*-methyl-6-hydroxy-apigenin	*Araucaria bidwilli*	[68]
8,11,13-abietatrien-15-ol	*Agathis macrophylla*	[16]
(13*S*)-pimar-7-en-3α,15,16-triol	*Agathis macrophylla*	[18]
13-*epi*-cupressic acid	*Araucaria heterophylla*	[86]
13-*O*-acetyl-13-*epi*-cupressic acid	*Araucaria heterophylla*	[86]
13-oxo-podocarp-8(14)-en-19-oate	*Agathis macrophylla*	[16]
15,19-diacetoxylabd-8(17)-en	*Araucaria araucana*	[61]
15ξ-hydroxy-pinusolidic acid	*Agathis macrophylla*	[16]
15-acetoxylabd-8(17)-en-19-ol	*Araucaria araucana*	[61]
15-acetoxy-imbricatolal	*Araucaria araucana*	[59,61]
15-acetoxy- imbricatolic acid	*Araucaria araucana*	[59,60,61]
15-formyloxy-imbricatolal	*Araucaria araucana*, *Wollemia nobilis*	[31,59]
15-formyloxy-imbricatolicacid	*Wollemia nobilis*	[31]
15-hydroxy-imbricatolal	*Araucaria araucana*	[59,60,61]
15-hydroxy-imbricatolic acid	*Araucaria araucana*	[59]
15-*nor*-14-oxolabda-8(17),12*E*-dien-19-oicacid, 13-oxo-podocarp-8(14)-en-19-oic acid	*Agathis macrophylla*	[16]
16-hydroxy-8(17),13-labdadien-15,16-olid-19-oic acid	*Agathis macrophylla*	[16]
19-hydroxylabd-8(17)-en-15-oic acid	*Araucaria araucana*	[61]
19-noranticopalic acid	*Agathis lanceolata*	[14]
α-linolenic acid	*Agathis robusta*	[25]
(−)-*epi*-afzelechin *p*-hydroxybenzoate	*Araucaria angustifolia*	[46]
(−)-*epi*-afzelechin protocatechuate	*Araucaria angustifolia*	[46]
(−)-*epi*-catechin	*Araucaria angustifolia*	[46,55]
(−)*seco*-isolariciresinol	*Araucaria angustifolia*	[54]
β-sitosterol	*Agathis macrophylla*, *Araucaria angustifolia*, *Araucaria columnaris*	[17,44,75]
β-sitosterol-3-*O*-glucopyranoside	*Araucaria bidwilli*	[69]
β-sitosterol acetate	*Araucaria columnaris*	[75]
abietic acid	*Araucaria columnaris*, *Agathis macrophylla*	[18,19,75]
acetyl-isocupressic acid	*Wollemia nobilis*	[28,29,30,32,33]
agatharesinol	*Agathis macrophylla*	[18]
agathic acid	*Agathis macrophylla, Agathis microstachya*, *Araucaria angustifolia*, *Wollemia nobilis*	[18,19,20,29,32,44]
agathic acid dimethyl ester	*Araucaria columnaris*	[75]
agathisflavone	*Agathis alba*, *Agathis atropurpurea*, *Agathis australis*, *Agathis ovata*, *Agathis robusta*, *Araucaria bidwilli*, *Araucaria columnaris*, *Araucaria rulei*, *Wollemia nobilis*	[6,9,22,31,70,89]
agatholic acid	*Agathis lanceolata*, *Araucaria angustifolia*, *Araucaria araucana*	[15,44,61]
alanine	*Wollemia nobilis*	[29,33]
amentoflavone	*Agathis macrophylla*, *Araucaria angustifolia,* *Araucaria bidwilli*, *Araucaria columnaris*, *Araucaria rulei*	[6,17,52,53,70,89]
angustanoic acid F	*Agathis macrophylla*	[16]
apigenin	*Araucaria angustifolia*	[45]
arachidonic acid	*Agathis robusta*	[25]
arginine	*Wollemia nobilis*	[28]
benzoic acid	*Araucaria angustifolia*	[46]
bilobetin	*Agathis alba*, *Araucaria angustifolia*, *Araucaria bidwilli*	[6,56,70]
bishomolinoleic acid	*Agathis robusta*	[25]
bishomo-α-linolenic acid	*Agathis robusta*	[25]
cabreuvin	*Araucaria angustifolia*	[57]
caffeic acid	*Araucaria cunninghamii*, *Wollemia nobilis*	[31,32,82]
catechin	*Araucaria angustifolia*, *Araucaria columnaris*, *Araucaria cunninghamii*	[45,49,55,75,82]
catechol	*Agathis macrophylla*	[17]
*cis*-communic acid	*Agathis microstachya*	[20]
*cis*-vaccenic acid	*Agathis robusta*	[25]
chlorogenic acid	*Araucaria columnaris*, *Araucaria cunninghamii*	[75,82]
corchoionoside C	*Agathis macrophylla*	[17]
cupressuflavone	*Agathis robusta*, *Araucaria angustifolia*, *Araucaria bidwilli*, *Araucaria columnaris*, *Araucaria rulei*, *Wollemia nobilis*	[6,22,31,56,70,89]
D-lactic acid	*Wollemia nobilis*	[29,33]
dactylifric acid	*Wollemia nobilis*	[31]
dodecanoic acid	*Araucaria angustifolia*	[48]
ellagic acid	*Araucaria cunninghamii*	[82]
*ent*-8β,15-labd-*E*-13-ene-diol	*Araucaria bidwilli*	[72]
*en*t-l5-acetoxy-labda-8,*E*-13-diene	*Araucaria bidwilli*	[72]
*ent*-19-(*E*)-coumaroyloxy-labda-8(17),13(16),14-triene	*Araucaria cunninghamii*	[79]
*ent*-19-(*Z*)-coumaroyloxy-labda-8(17),13(16),14-triene	*Araucaria cunninghamii*	[79]
*ent*-labda-8,*E*-13-dien-15-ol	*Araucaria bidwilli*	[72]
*epi*-catechin	*Araucaria angustifolia*, *Araucaria cunninghamii*	[45,49,82]
*epi*-pinoresinol	*Araucaria angustifolia*	[58]
eriodictyol-*O*-hexoside	*Araucaria angustifolia*	[55]
eudesmin	*Araucaria angustifolia*, *Araucaria araucana*	[44,50,51,54,57,58,62]
ferruginol	*Araucaria angustifolia*	[58]
ferulic acid	*Araucaria columnaris*	[75]
ferulic acid hexoside	*Araucaria angustifolia*	[55]
gallic acid	*Araucaria columnaris*, *Araucaria cunninghamii*	[73,75,82]
ginkgetin	*Araucaria angustifolia*	[52,53]
glucose	*Wollemia nobilis*	[28,29,30,33]
hexadecanoic acid	*Araucaria angustifolia*	[48]
hinokiflavone	*Araucaria bidwilli*, *Araucaria columnaris*, *Araucaria cunninghamii*	[6,70]
hinokiresinol	*Araucaria angustifolia*	[54,58]
hydroquinone	*Araucaria angustifolia*	[58]
imbricatolic acid	*Araucaria angustifolia*, *Araucaria araucana*	[44,60,61]
irisolidone	*Araucaria angustifolia*	[57]
*iso*-orientin	*Araucaria columnaris*	[73]
*iso*-vitexin	*Araucaria columnaris*	[73]
isocupressic acid	*Wollemia nobilis*	[28,29,32]
isolariciresinol	*Araucaria angustifolia*	[50,51,54,58]
isolariciresinol-4′-methyl ether	*Araucaria angustifolia*	[50,51]
isolariciresinol-acetate	*Araucaria angustifolia*	[58]
junicedric acid	*Araucaria araucana*	[61]
kaempferol	*Araucaria cunninghamii*	[82]
kaur-16-en-3α,l3-diol	*Agathis macrophylla*	[18]
kauran-3α,l3,16a-triol	*Agathis macrophylla*	[18]
kayaflavone	*Araucaria cunninghamii*	[70]
kolavenic acid	*Araucaria bidwilli*	[67]
labda-8(14),15(16)-dien-3β-ol	*Araucaria cunninghamii*	[79]
labda-8(17),14-diene	*Araucaria heterophylla*	[86]
labd-8(17)-en-15,19-dial	*Araucaria araucana*	[61]
labda-8(20),13-dien-15-oic acid	*Araucaria bidwilli*	[67]
labda-8(20), 13-dien-15,19-dioic acid	*Araucaria bidwilli*	[67]
lambertianic acid	*Agathis macrophylla*	[16]
lariciresinol	*Araucaria angustifolia*, *Araucaria araucana*	[50,51,57,58,62]
lariciresinol-4,4′-dimethyl ether-9-acetate	*Araucaria angustifolia*	[58]
lariciresinol-4-methyl ether	*Araucaria angustifolia*, *Araucaria araucana*	[58,62]
lariciresinol-4′-methyl ether	*Araucaria angustifolia*	[58]
lariciresinol-4-methyl ether-9-acetate	*Araucaria angustifolia*	[58]
lariciresinol-9-acetate	*Araucaria angustifolia*	[58]
linoleic acid	*Agathis robusta*	[25]
luteolin	*Araucaria columnaris*	[75]
methyl *ent*-8α-hydroxy-labd-*E*-l3-en-15-oate	*Araucaria bidwilli*	[72]
methyl *ent*-8β-hydroxy-labd-*E*-l3-en-15-oate	*Araucaria bidwilli*	[72]
methyl-15-hydroxy-abietate	*Agathis microstachya*	[20]
methyl-15-hydroxy-dehydroabietate	*Agathis microstachya*	[20]
methyl-communate	*Araucaria columnaris*	[75]
methyl-(*E*)-communate	*Wollemia nobilis*	[28,33]
methyl abietate	*Agathis microstachya*	[20]
methyl lambertianate	*Agathis macrophylla*	[16]
methyl sandaracopimarate	*Agathis lanceolata*, *Agathis microstachya*	[15,20]
myricetin	*Araucaria columnaris*	[75]
nyasol	*Araucaria angustifolia*	[58]
*neo*-abietic acid	*Agathis microstachya*	[20]
octadecyl-(*E*)-ferulate	*Araucaria angustifolia*	[57]
octadecyl-(*Z*)-ferulate	*Araucaria angustifolia*	[57]
octadecyl-(*E*)-*p*-coumarate	*Araucaria angustifolia*	[57]
octadecyl-(*Z*)-*p*-coumarate	*Araucaria angustifolia*	[57]
oleic acid	*Agathis robusta*	[25]
orientin	*Araucaria columnaris*	[73]
*p*-coumaric acid	*Araucaria angustifolia*	[58]
*p*-hydroxybenzoic acid	*Araucaria angustifolia*	[46]
pheophorbide *a*	*Wollemia nobilis*	[32]
phloretic acid	*Araucaria bidwilli*	[69]
pinitol	*Wollemia nobilis*	[29]
pinoresinol	*Araucaria angustifolia*, *Araucaria araucana*	[54,57,58,62]
pinoresinol monomethyl ether	*Araucaria angustifolia*	[54,58]
pinusolide	*Agathis macrophylla*	[16]
pinusolidic acid	*Agathis macrophylla*	[16]
prodelphinidin B	*Araucaria angustifolia*	[55]
protocatechuic acid	*Araucaria angustifolia*, *Wollemia nobilis*	[31,46,55]
quercetin	*Agathis macrophylla*, *Araucaria angustifolia*, *Araucaria columnaris*, *Araucaria cunninghamii*	[17,45,46,75,82]
quercetin-3-*O*-glucoside	*Araucaria angustifolia*	[55]
quinic acid	*Araucaria columnaris*, *Wollemia nobilis*	[29,33,80]
raffinose	*Wollemia nobilis*	[33]
robustaflavone	*Araucaria rulei*	[89]
rutin	*Araucaria angustifolia*, *Agathis robusta*, *Araucaria columnaris*	[22,49,75]
sandaracopimaradienol	*Agathis lanceolata*	[15]
sandaracopimaric acid	*Araucaria araucana*, *Wollemia nobilis*	[28,29,30,32,33,61]
*seco*-isolariciresinol	*Araucaria angustifolia*, *Araucaria araucana*	[50,51,58,62]
*seco*-isolariciresinol-4-methyl ether-9′-acetate	*Araucaria angustifolia*	[58]
*seco*-isolariciresinol-4-methyl ether-9,9′-diacetate	*Araucaria angustifolia*	[58]
*seco*-isolariciresinol-9′-acetate	*Araucaria angustifolia*	[58]
*seco*-isolariciresinol-9,9′-diacetate	*Araucaria angustifolia*	[58]
shikimic acid	*Agathis robusta*, *Wollemia nobilis*	[22,28,29,30,32,33]
shikimic acid *n*-butyl ester	*Araucaria cunninghamii*	[79]
shonanin	*Araucaria angustifolia*	[58]
sitosterol	*Agathis macrophylla*	[18]
stigmastan-3,5-diene	*Araucaria columnaris*	[75]
succinic acid	*Wollemia nobilis*	[33]
sucrose	*Wollemia nobilis*	[28,29,33]
sugiol	*Araucaria angustifolia*	[44]
taxifolin	*Araucaria columnaris*	[73]
taxifolin-3-*O*-glucopyranoside	*Araucaria columnaris*	[73]
tri-linolenoyl-*sn*-glycerol	*Wollemia nobilis*	[29]
*trans*-communic acid	*Agathis microstachya*, *Araucaria angustifolia*	[20,57]
umbelliferone	*Araucaria cunninghamii*	[82]
vanillic acid	*Araucaria columnaris*	[75]
vitexin	*Araucaria columnaris*	[73]
wollemol	*Wollemia nobilis*	[28,33]
wollemolide	*Wollemia nobilis*	[30,31]
